# Stimulator of Interferon Genes (STING) Promotes *Staphylococcus aureus*-Induced Extracellular Traps Formation via the ROS-ERK Signaling Pathway

**DOI:** 10.3389/fcell.2022.836880

**Published:** 2022-03-23

**Authors:** Zhen-Zhen Liu, Wei Chen, Cheng-Kai Zhou, Ke Ma, Yu Gao, Yong-Jun Yang

**Affiliations:** Key Laboratory of Zoonosis Research, Ministry of Education, College of Veterinary Medicine, Jilin University, Changchun, China

**Keywords:** STING, *Staphylococcus aureus*, extracellular traps, NADPH oxidase, innate immune

## Abstract

Stimulator of interferon genes (STING) is a cytosolic DNA sensor or directly recognizes bacterial cyclic dinucleotides, which is required for the detection of microbial infection. Extracellular traps (ETs) are known to be part of the antimicrobial defense system. However, the implication of STING in ETs formation during microbial infection remains unknown. Here, we showed that STING contributed to *Staphylococcus aureus* (*S. aureus*)-induced ETs formation through the ROS-ERK signaling. STING deficiency exhibited decreased cell-free DNA (cfDNA) level, reduced expression of citrullinated histone H3 (CitH3), and diminished DNA colocalization with CitH3 and myeloperoxidase (MPO). Interestingly, NADPH oxidase-derived reactive oxygen species (ROS) promoted ETs formation, accompanied by increased activation of extracellular signal-regulated kinase 1 and 2 (ERK1/2) in *S. aureus*-stimulated bone marrow-derived macrophages (BMDMs). Corresponding to less ROS production, decreased ERK1/2 activation was shown in STING^-/-^ BMDMs after *S. aureus* infection. Importantly, inhibiting the ROS-ERK signal reduced the ETs formation and the differences disappeared between WT and STING^-/-^ BMDMs after *S. aureus* infection. Moreover, STING^-/-^ BMDMs exhibited significantly increased levels of extracellular bacteria compared to WT BMDMs regardless of phagocytosis. In addition, such differences disappeared after DNase I treatment. DNase I treatment also facilitated pathogen colonization without affecting the inflammatory cells infiltration and pro-inflammatory factors secretion following pulmonary *S. aureus* infection. Furthermore, STING^-/-^ mice presented decreased levels of cfDNA and CitH3, along with increased bacterial colonization compared to WT mice. Altogether, these findings highlighted that STING promoted ETs formation via the ROS-ERK signal for host defense against *S. aureus* infection.

## Introduction

*Staphylococcus aureus* (*S. aureus*) is not only a symbiotic bacterium persistently carried by both humans and domestic animals but also an opportunistic pathogen posing a threat to public health and agriculture (Lowy, 1998; Vanbelkum et al., 2009). Once the delicate balance between *S. aureus* and the host is disturbed, *S. aureus* can display its pathogenic potential, which relates to an extensive range of infections including pneumonia, meningitis, osteomyelitis, endocarditis, and toxic shock syndrome (Stapleton and Taylor, 2002). The emergence and rapid spread of methicillin-resistant *Staphylococcus aureus* (MRSA) has become a major focus for infection control efforts. Vancomycin is used as the last line of defense against *S. aureus* infection. However, resistance against this antibiotic is on the rise in some *S. aureus* strains, and no effective vaccine is currently available (Miller et al., 2020). Hence, understanding the interaction mechanism between host and pathogen is a prerequisite for designing successfully therapeutic strategies.

Recently, extracellular traps (ETs) have been characterized as a novel extracellular protective defense mechanism against various pathogens, a process is known as ETosis which is a mode of cell death that is different from necrosis or apoptosis (Fuchs et al., 2007). These weblike structures consist of DNA fibers and antimicrobial proteins, including histones, antimicrobial peptides, and proteases. These structures have been described for the first time in neutrophils as termed NETs by Brinkmann *et al* (Brinkmann et al., 2004). Similar phenomena were also reported in basophils, eosinophils, mast cells, monocytes, as well as macrophages (Aulik et al., 2012; von Köckritz-Blickwede et al., 2008; Yousefi et al., 2008; Schorn et al., 2012). ETs could capture and kill microorganisms and have been associated with aseptic inflammation and autoimmune disease.

To date, studies have shown that ETs formation is different by a variety of stimuli through distinct mechanisms (Burgener and Schroder, 2020). NETs formation is mainly classified into three models reported to date. In the classic model, PMA can be exemplified for NADPH oxidase-dependent suicidal NETosis, which leads to reactive oxygen species (ROS) production, myeloperoxidase (MPO) activation, neutrophil elastase (NE) release, chromatin decondensation, and the release of cytoplasmic contents into the extracellular space via GSDMD (Gasdermin D) pores (Sollberger et al., 2018). In contrast to suicidal NETosis, vital NETosis induces rapid NETs release that is dispensable of NADPH oxidase. Enhanced intracellular calcium signaling activates peptidylarginine deiminase 4 (PAD4) to trigger histone citrullination and DNA decondensation which represents the most specific biomarker for vital NETosis. For example, *Shigella flexneri*-infected neutrophils undergone vital NETosis in a PAD4-dependent manner (Li et al., 2010). The third type of NETosis is termed noncanonical NETosis, which needs the participation of caspase-4/11 and GSDMD. PAD4-induced histone citrullination occurred in the process of noncanonical NETosis but was not necessary for cell death or NET extrusion (Chen et al., 2018).

Innate immunity is crucial in the early recognition and defense against multiple pathogens. Highly evolved conservative components from pathogens are recognized by pattern recognition receptors (PRRs) of the host cells (Kaufmann and Dorhoi, 2016). Stimulator of interferon genes (STING) acts as a key cytosolic DNA sensor. STING’s function has been extensively studied on its induction of type I interferons (IFNs) (Ma and Damania, 2016). To our knowledge, only one study has reported that STING participated in NETs formation induced by mitochondrial DNA (mtDNA) (Liu et al., 2020). However, the role of STING in bacteria-induced ETs formation has been poorly investigated.

In our study, we showed that STING facilitated the formation of ETs following *S. aureus* infection. STING deficiency exhibited decreased cell-free DNA (cfDNA) release, diminished expression of citrullinated histone H3 (CitH3), and reduced ETs structures. More importantly, STING deficiency led to a defect in ETs formation owing to the impaired ROS-ERK signaling pathway. As expected, STING-mediated ETs formation enhanced host defense against *S. aureus* infection by improving the extracellular killing capacity. Collectively, these findings suggested that STING was essential for *S. aureus*-induced ETs formation through the ROS-ERK signaling pathway.

## Materials and Methods

### Mice

STING^-/-^ mice (Jackson Laboratory) were backcrossed for >8 generations with C57/BL/6J mice. Sex- and age-matched (6–8 weeks) wild-type (WT) controls were used for all the experiments. Mice were maintained on a standard laboratory chow diet and water ad libitum. Procedures were approved by the Animal Welfare and Research Ethics Committee at Jilin University (No. 20150601).

### Pneumonia Model

The mouse pneumonia model was performed as we previously described (Liu et al., 2021). Briefly, mice were anesthetized intraperitoneally (*i.p.*) with pentobarbital sodium (50 mg/kg) and then infected intranasally (*i.t.*) with 20 μL volume of *S. aureus* strain USA300-TCH1516 (1 × 10^8^ CFU/mouse). At 6 h or 24 h post infection (hpi), bronchoalveolar lavage fluid (BALF) was obtained by perfusing the lungs with phosphate buffered saline (PBS) for three times. Lungs were collected following BALF collection. Serial dilutions of both the BALF and lung homogenates were plated on tryptic soy broth (TSB) agar for quantification of bacterial burden. In other experiments, mice were treated *i. t.* with DNase I (4000 U/mouse, Yuanye Bio-Technology, Shanghai, China) every 8 h. BALF leukocytes were counted using a hemocytometer. We quantified protein content by ELISA for albumin (ML720151, Mlbio, Shanghai, China) detection according to instructions.

### Cytokine Measurements

The expression levels of IL-1β, IL-6, and TNF-α in BALF and lung tissue homogenates were measured using the ELISA system according to the manuals. The ELISA kits were obtained from R&D Systems.

### Cell Culture and Cell Infection

To prepare BMDMs, bone marrow cells were collected from femurs of mice (6-8-week-old) and then treated with erythrocyte-lysing buffer to remove red blood cells. The collected cells were cultured in RPMI 1640 (Gibco, Waltham, MA, United States) containing 25% L929 cell-conditioned medium for 7 days. Bone marrow-derived neutrophils (BMDNs) were isolated using the mouse neutrophil isolation kit (TBD2012NM, Haoyang, Tianjin, China). BMDMs or BMDNs were infected with *S. aureus* at a multiplicity of infection (MOI) of 20. At the indicated time points, the supernatants and cell lysates were collected, clarified, and analyzed by ETs quantitation or Western blotting assays.

### Inhibition Assay

BMDMs were separately treated with an equal volume of DMSO, 10 µM of extracellular signal-regulated kinase 1 and 2 (ERK1/2) inhibitor U0126 (Sigma Aldrich, St. Louis, MO, United States), or 10 µM of NADPH oxidase inhibitor DPI (Sigma Aldrich) for 30 min before *S. aureus* (MOI: 20) stimulation. At 3 hpi, the samples were collected for corresponding assays.

### ROS Detection Assay

WT and STING^-/-^ BMDMs were infected with *S. aureus* at an MOI of 20 for 3 h. Then added 10 µM 2,7-dichlorofluorescin-diacetate (DCFH-DA, S0033S, Biyuntian, China), and the BMDMs were incubated at 37°C for 20 min in the dark. The plates were washed three times with RPMI 1640 to remove excess extracellular DCFH-DA. The samples were examined with a fluorometric reader (BioTek, United States). The excitation and emission wavelengths were 485 and 530 nm, respectively.

### Quantitation of Extracellular Traps

The released ETs were quantified by analyzing the SYTOX Green intensity as described previously (Li et al., 2018). BMDMs or BMDNs obtained from WT and STING^-/-^ mice were infected with *S. aureus* at an MOI of 20 for designed time points. In parallel experiments, before infection with *S. aureus*, BMDMs were pretreated with the inhibitors as described above. Besides, DNase I (100 U/mL) was used to eliminate the ETs structure for 20 min before *S. aureus* infection. The samples of culture supernatants were collected and centrifuged. A volume of 90 μL of each supernatant was transferred into 96-well plates. The cfDNA was stained with 5 μM SYTOX Green (Invitrogen, Carlsbad, CA, United States) and then quantified by a fluorescent reader. The excitation and emission wavelengths were 485 and 530 nm, respectively.

### Extracellular Bacterial Killing Assay

WT and STING^-/-^ BMDMs were infected with *S. aureus* at an MOI of 20 for 3 h. In parallel experiments, before infection with *S. aureus*, BMDMs were pretreated with cytochalasin D (CytD, 10 μM, Sigma Aldrich) for 20 min to inhibit phagocytosis or DNase I (100 U/mL) for 20 min to eliminate the ETs structure before *S. aureus* infection. The samples of culture supernatants were collected, serially diluted, and cultured on TSB agar plates for 18 h at 37°C.

### Western Blotting Assay

BMDMs or lungs obtained from WT and STING^-/-^ mice were lysed in cold RIPA lysis buffer containing protease inhibitor cocktail (P8340, Sigma-Aldrich). Samples were centrifuged and supernatants were used for immunoblotting. Nuclear and cytosolic proteins were extracted and separated from BMDMs using a nuclear protein extraction kit (BB-3102, Bestbio, Shanghai, China). Equal amounts of protein were resolved by 12% SDS-PAGE gels and transferred onto PVDF membranes. After blocking with 5% nonfat milk for 2 h, the membranes were incubated overnight with appropriate primary monoclonal antibodies against mouse p-ERK (9101, Cell Signaling Technology, Beverly, MA, United States), p-P38 (9211, Cell Signaling Technology), CitH3 (ab5103, Abcam, Cambridge, MA, United States), H3 (17168-1-AP, Proteintech, Wuhan, Hubei, China), p-STING (72971, Cell Signaling Technology) and GAPDH (10494-1-AP, Proteintech). The next day, membranes were washed and incubated with second antibodies at room temperature for 1 h, followed by ECL chemiluminescence reagent chromogenic.

### Fluorescence Confocal Microscopy Analyses

For *in vivo* experiments, BALF from *S. aureus*-infected mice was collected, centrifuged, and replated onto poly-L-lysine (0.1 mg/ml, Sigma-Aldrich) coated coverslips at 37°C for 1 h. For *in vitro* experiments, BMDMs or BMDNs were stimulated with *S. aureus* (MOI: 20, 3 h) on poly-l-lysine pre-coated coverslips. For *in vivo* or *in vitro* experiments, after incubation, the cells were fixed with 4% paraformaldehyde for 20 min, permeabilized with 0.1% Triton X-100 for 20 min, and washed with PBS for 10 min. After blocking with 5% goat serum for 1 h, the cells were incubated with monoclonal antibodies to CitH3 (ab5103, Abcam, 1:200) and MPO (ab9535, Abcam, 1:100) at 4°C overnight. Then, the cells were incubated with the CoraLite594-conjugated secondary antibody (SA00013-4, Proteintech) and stained with 5 μM SYTOX green for 10 min at room temperature. Finally, the specimens were mounted by anti-fluorescence quenching sealing tablets (Beyotime Biotechnology, China) and observed using fluorescence microscopy or laser confocal microscopy.

### Statistical Analysis

Data are represented as the mean ± SEM. The statistical comparisons were performed using a one-way ANOVA with Dunnett’s multiple comparison test among multiple groups with a single control and two-way ANOVA with Bonferroni’s multiple comparison test among different groups. *p* values less than 0.05 (*p < 0.05, **p < 0.01, ***p < 0.001) were considered to be statistically significant.

## Results

### STING Facilitates *S. aureus*-Induced ETs Formation *in vitro*

**FIGURE 1:**
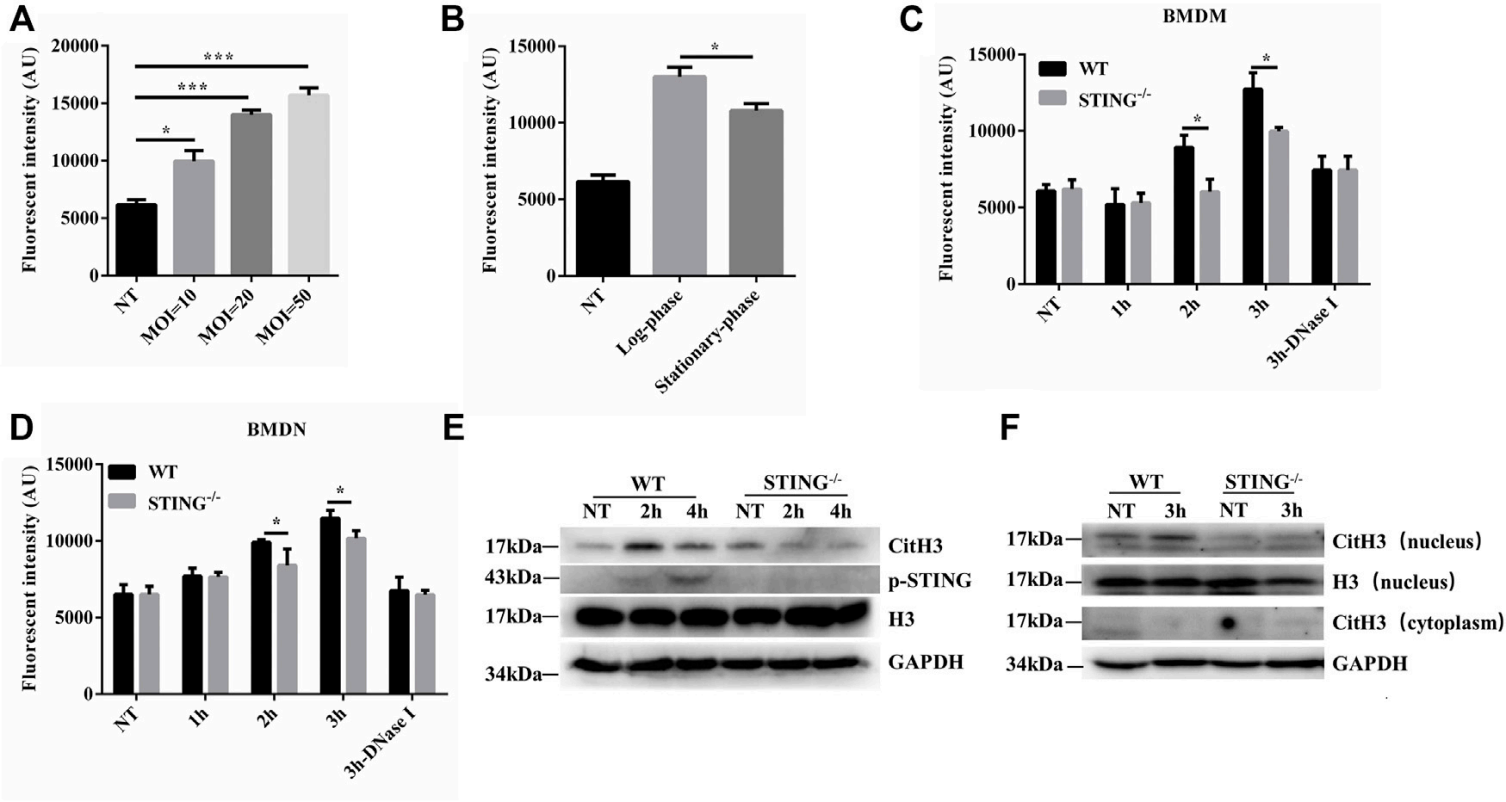
STING promotes ETs formation in macrophages and neutrophils after *S. aureus* infection. WT BMDMs were infected with *S. aureus* at designed MOIs and different periods of growth. **(A,B)** The cfDNA in supernatants was stained with SYTOX Green and analyzed on a fluorescent reader. BMDMs or BMDNs from WT and STING^-/-^ mice were infected with *S. aureus* (MOI: 20) at the designed time points. **(C,D)** Cells from WT and STING^-/-^ mice were treated with DNase Ⅰ (100 U/mL) 20 min before infection with *S. aureus*. The cfDNA in supernatants was stained with SYTOX Green and analyzed on a fluorescent reader. **(E)** Whole cell lysates and **(F)** nuclear proteins were analyzed for CitH3, H3, p-STING, and GAPDH by Western blotting. Data are shown as mean ± SEM. Data were pooled from 3 independent experiments. Data were analyzed using two-way ANOVA with Bonferroni’s multiple comparison test. For all experiments in this figure: *p < 0.05.

**FIGURE 2:**
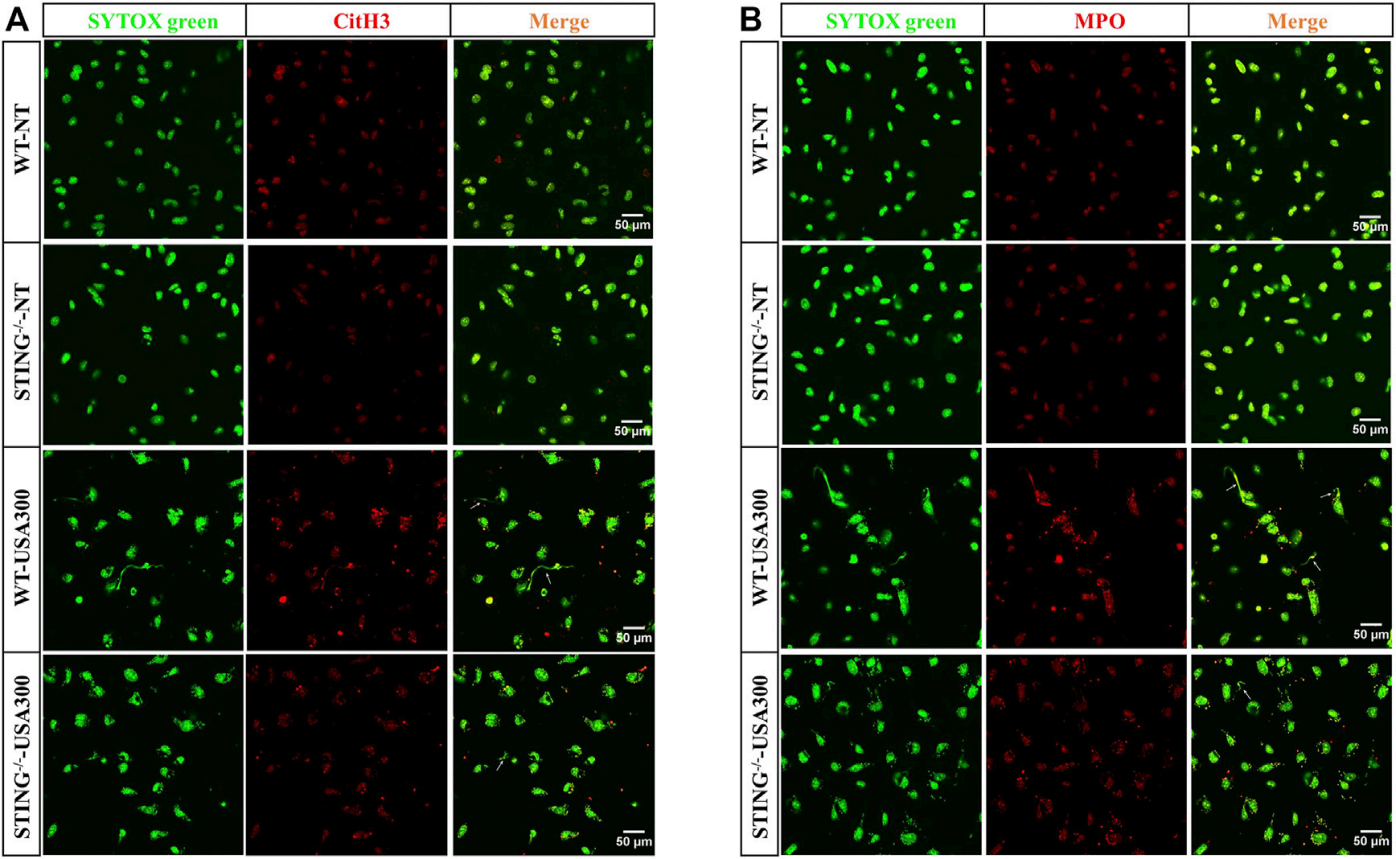
STING^-/−^ BMDMs present decreased colocalization of DNA decorated with CitH3 and MPO in *S. aureus*-induced ETs structures. WT and STING^-/-^ BMDMs were infected with *S. aureus* (MOI: 20) for 3 h and then fixed with 4% paraformaldehyde. DNA decorated with CitH3 **(A)** and MPO **(B)** within ETs were detected using a scanning confocal microscope. DNA was stained with SYTOX green. METs structures were marked with white arrows. The experiments were repeated 3 independent times.

In order to investigate ETs formation, WT BMDMs were determined the cfDNA levels after infection with *S. aureus* at various growth phases and different MOIs. The cfDNA release was increased at higher MOI values (Figure 1A). Compared to the log-phase, a decrease of cfDNA level was observed in the stationary-phase after *S. aureus* infection (Figure 1B). Therefore, to avoid the interference of other elements during ETs detection, the log-phase bacteria (MOI: 20) was used for subsequent studies. To investigate the impact of STING on ETs formation, we measured the cfDNA in supernatants following *S. aureus* infection by SYTOX green staining. Interestingly, the cfDNA level was distinctly decreased in STING^-/-^ BMDMs relative to WT BMDMs (Figure 1C). Further, BMDNs were infected with *S. aureus* and we also observed a decreased level of cfDNA in STING^-/-^ BMDNs compared to WT BMDNs (Figure 1D). Besides, we measured the expression of CitH3, marked CitH3 induction was observed both in total protein lysate and nuclear protein lysate after *S. aureus* stimulation. In line with the results described above, STING^-/-^ BMDMs exhibited significantly decreased levels of CitH3 compared to WT BMDMs (Figures 1E,F). Meanwhile, p-STING protein levels were significantly up-regulated by bacteria challenge in WT BMDMs and absent from STING^-/-^ BMDMs (Figure 1E). We set out to further characterize the colocalization of DNA and relevant proteins (CitH3 and MPO) by immunofluorescence (Fuchs et al., 2007). The results showed that STING^-/-^ BMDMs or BMDNs presented less colocalization of DNA decorated with these proteins after *S. aureus* infection (Figure 2, Supplementary Figure S1). Taken together, these findings suggested that STING contributed to *S. aureus*-induced ETs formation.

### NADPH Oxidase-Derived ROS Contributes to *S. aureus*-Induced ETs Formation

**FIGURE 3:**
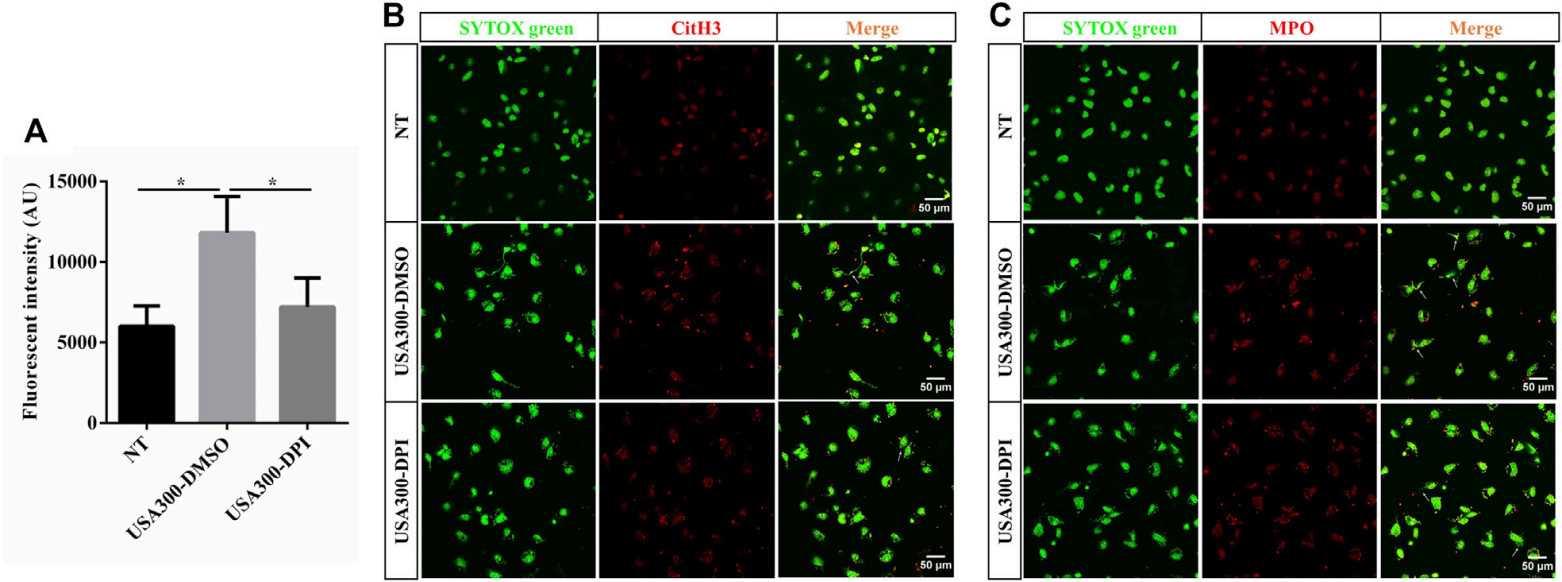
NADPH oxidase-derived ROS facilitates the *S. aureus*-induced ETs formation. WT BMDMs were pretreated with DPI (10 μM) and then infected with *S. aureus* (MOI: 20) for 3 h. **(A)** The cfDNA in supernatants was stained with SYTOX Green and analyzed on a fluorescent reader. DNA decorated with CitH3 **(B)** and MPO **(C)** within ETs were detected using a scanning confocal microscope. DNA was stained with SYTOX green. METs structures were marked with white arrows. Data were pooled from 3 independent experiments. Data were analyzed using one-way ANOVA with Dunnett’s multiple comparison test, *p < 0.05.

To explore whether *S. aureus*-induced ETs formation depends on NADPH oxidase-derived ROS, specific inhibitor DPI was used and ETs generation was assayed. When DPI was added, *S. aureus*-induced cfDNA was significantly decreased (Figure 3A). The colocalization of extracellular DNA with CitH3 and MPO was also suppressed with the addition of DPI (Figures 3B,C). These findings concluded that NADPH oxidase-derived ROS contributed to *S. aureus*-induced ETs formation.

### STING Promotes *S. aureus*-Induced Extracellular Traps Formation Depending on the Reactive Oxygen Species-Extracellular Signal-Regulated Kinase Signaling Pathway

**FIGURE 4:**
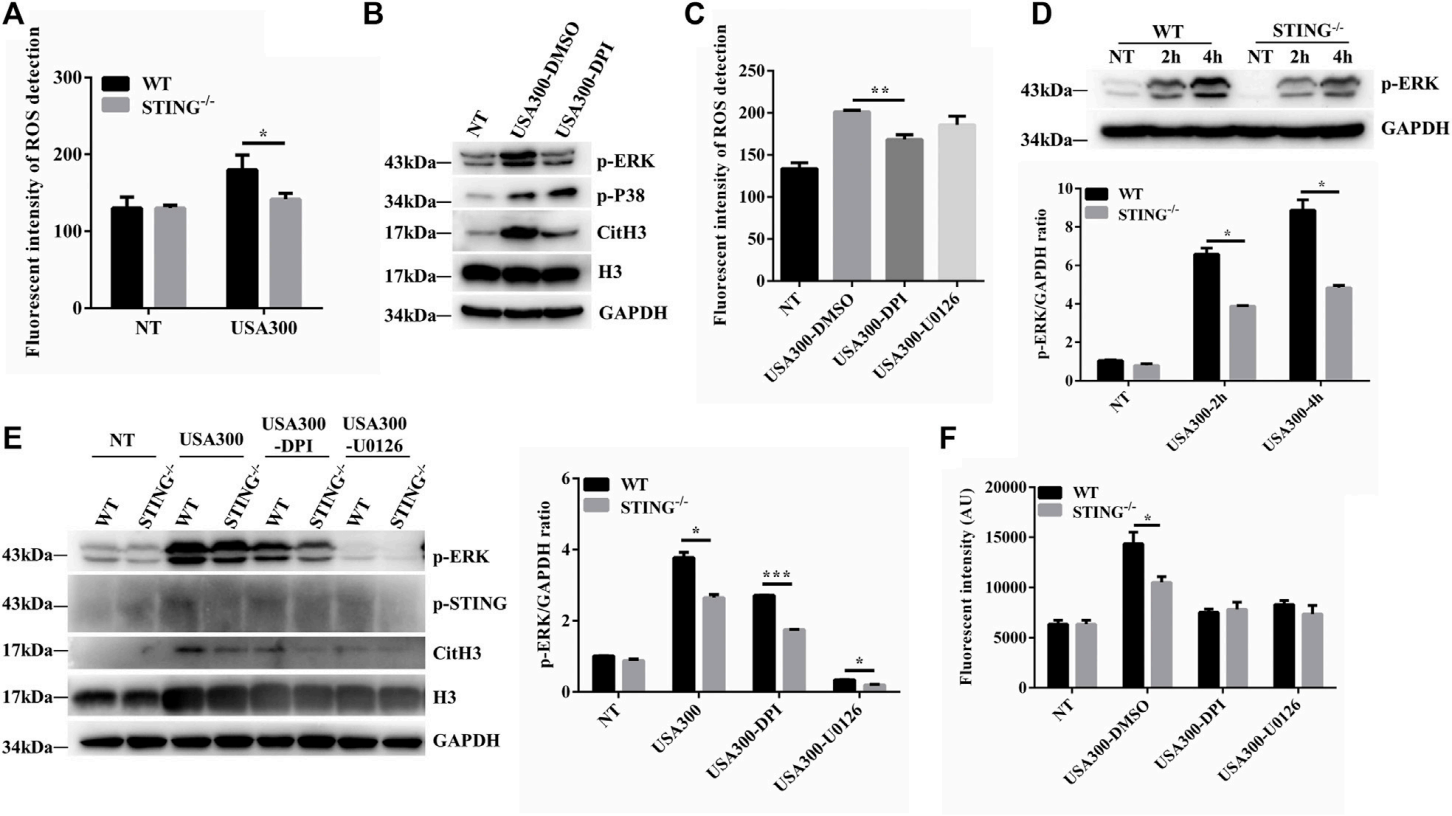
STING promotes ETs formation in a ROS-ERK-dependent manner upon *S. aureus* challenge. **(A)** Measurement of ROS production in BMDMs from WT and STING^-/-^ mice after infection with *S. aureus* (MOI: 20) for 3 h **(B)** WT BMDMs were pretreated with DPI (10 μM), and the cells were then infected with *S. aureus* (MOI: 20) for 3 h. Whole cell lysates were analyzed for p-ERK, p-P38, CitH3, H3, and GAPDH by Western blotting. **(C)** WT BMDMs were pretreated with DPI (10 μM) or U0126 (10 μM) for 1 h before *S. aureus* (MOI: 20) treatment for 3 h. ROS production was detected by DCFH-DA. **(D)** BMDMs from WT and STING^-/-^ mice were infected with *S. aureus* (MOI: 20) at the designed time points. Whole cell lysates were analyzed for p-ERK and GAPDH by Western blotting, and the gray intensity value was calculated using ImageJ software. WT and STING^-/-^ BMDMs were pretreated with DPI (10 μM) or U0126 (10 μM) for 1 h before *S. aureus* (MOI: 20) treatment for 3 h. **(E)** Whole cell lysates were analyzed for p-ERK, CitH3, H3, p-STING, and GAPDH by Western blotting, and the gray intensity value of p-ERK was calculated using ImageJ software. **(F)** The cfDNA in supernatants was stained with SYTOX Green and analyzed on a fluorescent reader. Data are shown as the mean ± SEM. Data were pooled from 3 independent experiments. Significance calculated using two-way ANOVA with Bonferroni’s multiple comparison test, *p < 0.05.

We further characterized the ROS production in BMDMs derived from WT and STING^-/-^ mice. Surprisingly, STING^-/-^ BMDMs produced less ROS than WT BMDMs after *S. aureus* infection (Figure 4A). NADPH oxidase-derived ROS activate the p38 MAPK and ERK1/2 signaling pathways (Ma et al., 2018). To determine the role of ROS following *S. aureus* stimulation, WT BMDMs were pretreated with DPI, and phosphorylation levels of p38 and ERK were analyzed by Western blotting. Importantly, after pretreatment with DPI, there was no significant change of p38 MAPK activation whereas ERK1/2 signal was inhibited after *S. aureus* infection (Figure 4B). DPI inhibited *S. aureus*-derived ROS whereas U0126 did not inhibit ROS production (Figure 4C). Meanwhile, we found that the levels of p-ERK were markedly decreased in *S. aureus*-infected STING^-/-^ BMDMs compared to WT BMDMs (Figure 4D). In order to confirm that the STING-regulated ROS-ERK signaling pathway participated in *S. aureus*-triggered ETs formation, U0126 or DPI was applied to analyze the expression of CitH3 and cfDNA release between WT and STING^-/-^ BMDMs. The results showed that U0126 or DPI treatment resulted in significant inhibition of *S. aureus*-induced expression of CitH3, coupled with the decreased expression of p-ERK. U0126 or DPI treatment had no effect on p-STING protein expression (Figure 4E). Consistent with the above results, the difference of cfDNA release disappeared after the addition of U0126 or DPI between WT and STING^-/-^ BMDMs challenged with *S. aureus* (Figure 4F). Collectively, these results indicated that STING deficiency led to a defect in ETs formation by impairing the ROS-ERK signaling pathway.

### STING Deficiency Reduces Extracellular Traps-Mediated Extracellular Killing Capacity by *S. aureus* Infection

**FIGURE 5:**
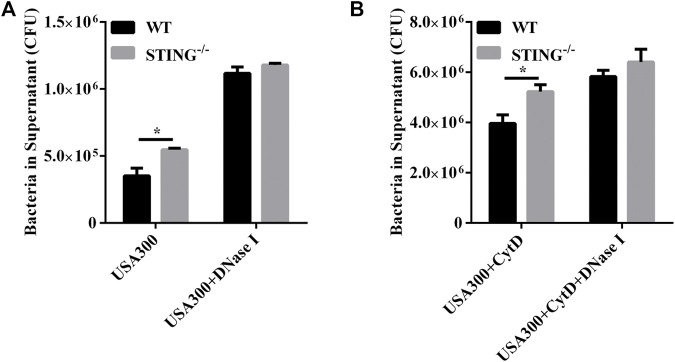
STING promotes ET-mediated extracellular bacterial killing by *S. aureus* infection. **(A,B)** WT and STING^-/-^ BMDMs were pretreated with DNase I (100 U/mL) or CytD (10 μM) for 20 min before infection with *S. aureus* (MOI: 20) for 3 h. The supernatants were collected and diluted, and the number of extracellular bacteria was determined. Data are shown as the mean ± SEM. Data were pooled from 3 independent experiments. Significance calculated using two-way ANOVA with Bonferroni’s multiple comparison test, *p < 0.05.

To further determine whether STING-mediated extracellular structures could kill the entrapped *S. aureus*, WT and STING^-/-^ BMDMs were infected with *S. aureus*, and the killing ability was identified by counting the number of extracellular bacteria. The results showed that STING^-/-^ BMDMs presented a marked increased number of extracellular bacteria compared to WT BMDMs. Simultaneously, we specifically used DNase I to degrade ETs and we observed a significant increase in the number of extracellular bacteria after DNase I treatment. Meanwhile, we found that the difference of extracellular bacterial load disappeared between WT and STING^-/-^ BMDMs in DNase I-treated groups (Figure 5A). In order to discriminate between bacteria killed by ETs or phagocytosis, phagocytosis was blocked by adding CytD. STING^-/-^ BMDMs still exhibited a significantly increased number of extracellular bacteria compared to WT BMDMs, such differences disappeared after DNase I treatment (Figure 5B). In conclusion, STING-mediated ETs improved host defense against *S. aureus* infection by enhancing the extracellular killing capacity.

### ETs Degradation With DNase I Is Detrimental During *S. aureus* Pulmonary Infection

**FIGURE 6:**
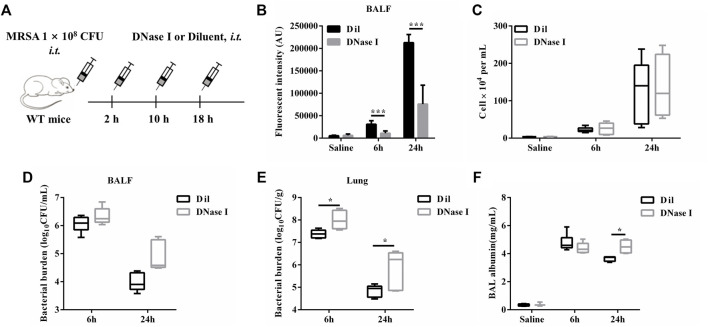
ETs degradation with DNase I is detrimental during *S. aureus* pulmonary infection. WT mice were challenged with *S. aureus* (1 × 10^8^ CFU/mouse, *i. t.*) and treated with DNase I (4000 U, *i. t.*) or diluent control at 2, 10, and 18 hpi (n = 4–6/group) and then were euthanized at 6 and 24 hpi. **(A)** A schema of the experiment timeline. **(B)** cfDNA in BALF, **(C)** BALF WBC, **(D)** bacterial counts in BALF, **(E)** bacterial counts in lungs, and **(F)** BALF albumin concentration were quantified. All data are shown as mean ± SEM. Data pooled from 2 independent experiments. Data were analyzed using two-way ANOVA with Bonferroni’s multiple comparison test, *p < 0.05, ***p < 0.001.

In order to investigate the role of ETs during *S. aureus* pulmonary infection. WT mice were treated *i. t.* with DNase I or diluent control at 2, 10, and 18 h after infection with *S. aureus* (Figure 6A). At 6 hpi or 24 hpi, we found that DNase I treatment effectively reduced the cfDNA level in BALF (Figure 6B) without affecting the infiltration of inflammatory cells (Figure 6C), as well as the release of pro-inflammatory cytokines (IL-1β, IL-6, and TNF-α) (Supplementary Figure S2). Moreover, a significantly increased bacterial load was observed in the lungs but not in BALF after DNase I treatment (Figures 6D,E). Since the addition of DNase I would affect the measurement of BALF total protein, we instead measured BALF albumin as an index of alveolar barrier dysfunction. The results showed that DNase I treatment induced significantly increased levels of BALF albumin at 24 hpi (Figure 6F). This may be attributed to the increased colonization of *S. aureus*. Collectively, these results showed that ETs were beneficial during pulmonary *S. aureus* infection.

### STING-Deficient Mice Present Decreased ETs Formation and Reduced Bacterial Clearance Following Pulmonary *S. aureus* Infection

**FIGURE 7:**
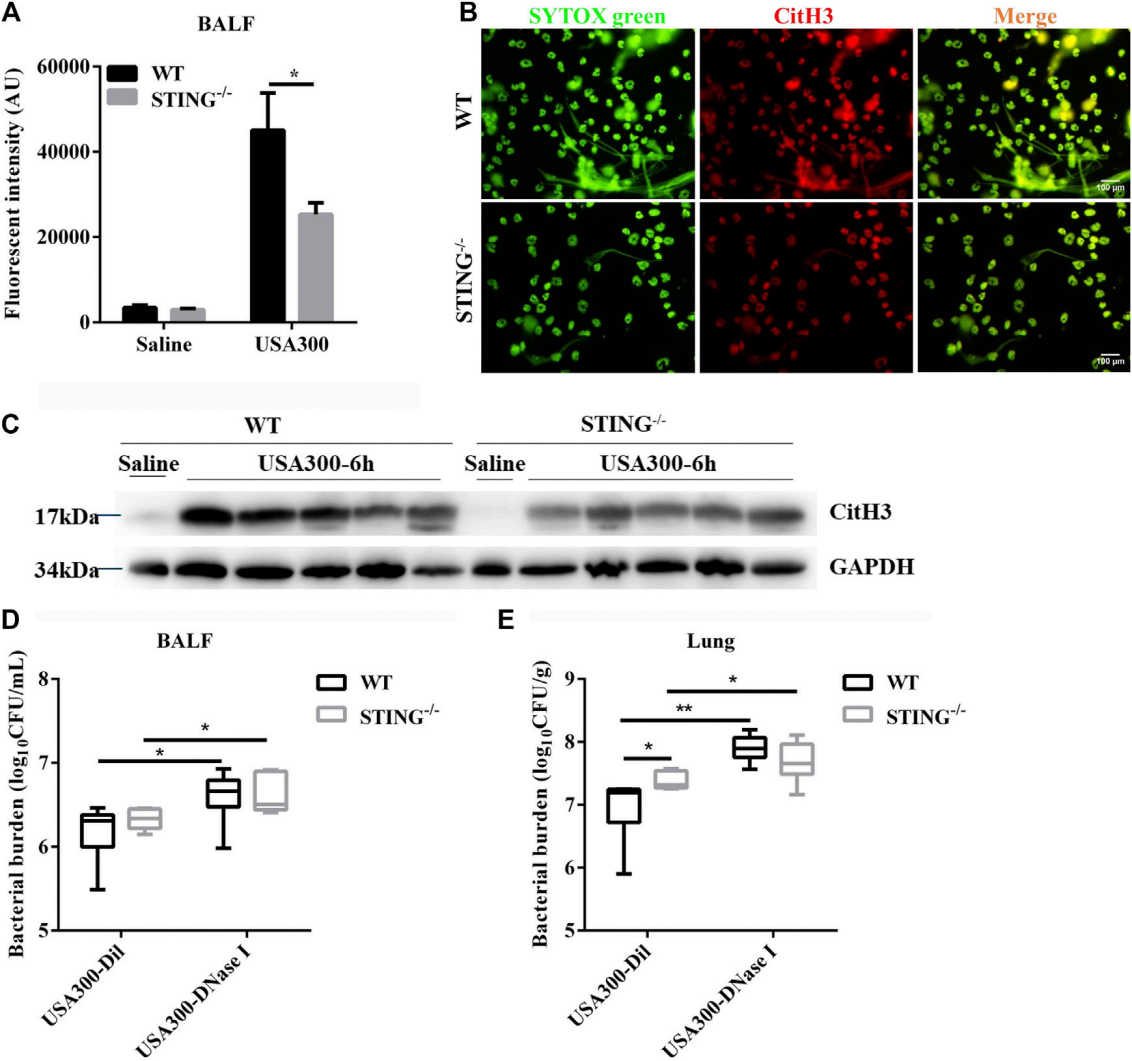
STING^-/−^ mice display decreased ETs production and reduced bactericidal ability during *S. aureus* pulmonary infection. WT and STING^-/-^ mice (n = 4–7/group) were infected with *S. aureus* (1 × 10^8^ CFU/mouse, *i. t.*) and samples were collected at 6 hpi. **(A)** cfDNA in BALF was quantified. **(B)** Cells of BALF were fixed *ex vivo*, DNA decorated with CitH3 was detected using a fluorescence microscope. DNA was stained with SYTOX green. **(C)** The lung tissue lysate was detected for the expression of CitH3 and GAPDH. WT and STING^-/-^ mice were challenged with *S. aureus* (1 × 10^8^ CFU/mouse, *i. t.*) and treated with DNase I (4000 U, *i. t.*) or diluent control at 2 hpi (n = 6–7/group) and then were euthanized at 6 hpi. **(D)** bacterial counts in BALF and **(E)** bacterial counts in lungs were quantified. All data are shown as mean ± SEM. Data pooled from 2 independent experiments. Data were analyzed using two-way ANOVA with Bonferroni’s multiple comparison test. For all experiments in this figure: *p < 0.05, **p < 0.01.

We further demonstrated the involvement of STING in ETs formation during pulmonary *S. aureus* infection. There may be a mutual cause-effect relationship between bacterial colonization and tissue damage. Therefore, we analyzed the initial host-bacteria interactions during the early phase of infection at 6 hpi. Our results showed that the cfDNA level in BALF from STING^-/-^ mice was significantly decreased compared to WT mice (Figure 7A). Meanwhile, we visualized the colocalization of extracellular DNA with CitH3 in collected cells of BALF from WT and STING^-/-^ mice. Utilizing fluorescence microscopy, we observed that STING deficiency significantly reduced ETs formation (Figure 7B). Furthermore, the expression of CitH3 in lung tissue was also markedly decreased in STING^-/-^ mice compared with WT mice (Figure 7C). Besides, a significant increase of bacteria load was observed in the lungs but not in BALF of STING^-/-^ mice compared to WT mice, such differences disappeared after DNase I treatment (Figures 7D,E). These findings suggested that STING-mediated ETs formation enhanced host resistance to *S. aureus* pneumonia.

## Discussion

*S. aureus* infection harms both human health and the development of the aquaculture industry. Understanding the mechanisms of host defense against *S. aureus* is necessary to design novel therapeutic strategies. ETs formation is a relatively novel antimicrobial mechanism for invasive pathogens independent of phagocytosis. The importance of ETs in innate immunity has been studied extensively, but the mechanism remains controversial and in scientific dispute during *S. aureus* infection. Several virulence factors of *S. aureus* were reported to elicit ETs formation, including leukotoxin (Malachowa et al., 2013), leukocidins (Bhattacharya et al., 2018), phenol-soluble modulin α (PSMα) (Björnsdottir et al., 2017), and protein A (Hoppenbrouwers et al., 2018). Importantly, *S. aureus* has also evolved mechanisms to avoid ET-based immune clearance, either through ETs degradation, resistance to the intrinsic antimicrobial effectors within ETs, or the suppression of ETs production. This is mainly attributed to the production of nuclease (Berends et al., 2010). *S. aureus* nuclease production was found to be associated with delayed bacterial clearance in the lung and significantly increased mortality during *S. aureus* respiratory tract infections (Wardenburg et al., 2007). Besides the bacterial own factors, the innate immune system is more essential for host protection against *S. aureus* infection.

STING is a PRR for cyclic dinucleotides and also functions as an adaptor protein for multiple cytosolic receptors of dsDNA. We demonstrated previously that IFI204 (a DNA sensor) is crucial for the host defense against *S. aureus* infection through promoting ETs formation (Chen et al., 2019). We have also previously shown that blocking necroptosis in STING^-/-^ mice possesses an intensified immune protection (Liu et al., 2021). There remains a significant gap between WT and STING^-/-^ mice, suggesting that STING may contribute to host defense against *S. aureus* pneumonia via additional mechanisms. This result can likely be attributed to the involvement of STING in *S. aureus*-induced ETs formation.

In this study, we uncovered a new mechanism by which STING promoted *S. aureus*-induced ETs formation. We first demonstrated that a significant reduction of extracellular DNA was seen in STING^-/-^ BMDMs or BMDNs compared with WT cells, as well as decreased expression of CitH3 in STING^-/-^ BMDMs compared with WT BMDMs. Some studies regarded CitH3 formation as an important biomarker for NETosis (Jin et al., 2014; Claushuis et al., 2018). However, other reports showed that CitH3 was not universally required for NETosis exposed to different stimuli (Kenny et al., 2017). Therefore, the relationship between ETs formation and histone H3 citrullination is concomitant rather than causal. NETs are net-like structures including a DNA-based scaffold with embedded histones and cellular proteins, including MPO, NE, and cathelicidin (Wartha et al., 2007; von Köckritz-Blickwede and Nizet, 2009; Papayannopoulos and Zychlinsky, 2009). In our study, the colocalization of DNA with CitH3 and MPO in *S. aureus*-induced ETs was significantly decreased in STING^-/-^ BMDMs compared to WT BMDMs. The above results indicated that STING serves an important function in *S. aureus*-induced ETs formation.

Previous studies have confirmed the effects of ROS production by NADPH oxidase in *S. aureus*-induced NETs formation (Fuchs et al., 2007). Thus, NADPH oxidase inhibitor DPI was performed to characterize the mechanisms of ROS in *S. aureus*-induced ETs formation in macrophages. Indeed, our results showed that DPI treatment reduced the formation of *S. aureus*-induced METs (macrophage extracellular traps), mainly characterized by decreased cfDNA level and diminished colocalization between DNA and relevant proteins (CitH3 and MPO). Hence, it suggested that *S. aureus*-induced ETs formation depended on the production of ROS. Previous studies have shown that NADPH oxidase-dependent ROS was involved in NETs formation induced by PMA and *Streptococcus Suis Serotype 2*, which then activated the ERK1/2 and p38 MAPK signaling pathways (Dusi et al., 1996; Keshari et al., 2013; Ma et al., 2018). Besides, neutrophils also experienced NADPH oxidase-dependent suicidal NETosis in a TLR4-JNK-dependent manner under LPS and Gram-negative bacteria stimulation (Khan et al., 2017). These studies indicated that ROS may function as an important second messenger to transmit diverse signals due to different stimuli. Moreover, STING has already been identified to participate in mtDNA‐induced NETs formation through p38 MAPK and ERK1/2 signals (Liu et al., 2020). Consequently, the relationship between ROS production and *S. aureus*-induced p38 MAPK or ERK1/2 activations was determined. In our study, ROS inhibition resulted in the suppression of ERK1/2 but not p38 MAPK. To clarify the role of STING in ROS-ERK mediated ETs formation, we detected the expression of p-ERK and ROS production. Compared with control cells, STING^-/-^ BMDMs exhibited less production of ROS and decreased levels of p-ERK. Importantly, inhibiting the ROS-ERK signal with inhibitors reduced the ETs formation and the differences disappeared between WT and STING^-/-^ BMDMs after *S. aureus* infection. Hence, our study emphasized the importance of STING in promoting *S. aureus*-induced ETs formation via the ROS-ERK signaling pathway.

The antimicrobial activity of ETs is mainly attributed to embedded histones and antimicrobial enzymes bound to the DNA backbone. To date, studies have shown that METs could catch and kill selected pathogens such as *S. aureus*, *S. agalactiae*, *E. coli*, and *C. albicans* (Liu et al., 2014; Vega et al., 2014; Bryukhin and Shopova, 2015; Shen et al., 2016). However, METs did not exhibit bactericidal capability on *M. abscessus*, instead of enhanced bacterial growth (Je et al., 2016). In our study, compared to WT BMDMs, STING^-/-^ BMDMs had a remarkably lower bactericidal capacity against extracellular *S. aureus*. Importantly, the differences in extracellular bacterial killing ability disappeared between WT and STING^-/-^ BMDMs after DNase I treatment. To distinguish the separate killing between MET-mediated and phagocytosis-mediated mechanisms, we used CytD to inhibit macrophage phagocytosis (Hellenbrand et al., 2013; Liu et al., 2014). STING^-/-^ BMDMs exhibited significantly increased extracellular bacteria burden compared to WT BMDMs pretreatment with CytD, such differences also disappeared after DNase I treatment. Hence, our results indicated that STING-mediated ETs formation enhanced phagocyte killing capacity.

ET is a double-edged sword. NETs have both protective and deleterious effects on lung infection and disease pathogenesis. Appropriate NETs contribute to control infection but excessive NETs formation lead to tissue injury (Gray, 2018). Previous study has demonstrated that digesting NETs with DNase I promoted bacterial colonization in WT mice during *Salmonella sifA* infection (Chen et al., 2018). Genetically knocking out PAD4 resulted in increased bacterial colonization and reduced survival (Lefrançais et al., 2018). These data are consistent with a previous study that highlighted that NETs could trap invasive pathogens to prevent bacterial dissemination (Beiter et al., 2006). Experiments presented in the current study demonstrated that DNase I treatment effectively reduced cfDNA in BALF. Importantly, DNase I treatment facilitated *S. aureus* colonization without affecting inflammatory cells infiltration and pro-inflammatory factors secretion. Consistent with this, STING^-/-^ mice presented decreased cfDNA level, diminished CitH3 expression, and reduced ETs structures compared to WT mice. Besides, STING^-/-^ mice exhibited higher bacterial loads in the lungs compared with WT mice, which further supported the role of STING-mediated ETs in bacterial clearance.

In summary, our study reveals the implication of STING in the regulation of *S. aureus*-induced ETs formation. STING deficiency exhibits lower extracellular DNA release, decreased expression of CitH3, and reduced ETs structures. Importantly, STING deficiency leads to a defect in ETs formation owing to the ROS-ERK signaling pathway. STING-mediated ETs improve host defense against *S. aureus* infection by enhancing the extracellular killing capacity *in vivo* and *in vitro*. These results uncover a potentially novel function of STING in host defense against extracellular bacterial infection.

## Data Availability

The original contributions presented in the study are included in the article/Supplementary Material, further inquiries can be directed to the corresponding author.

## Supplementary Material

Supplementary Figure 1 STING^-/-^ BMDNs present decreased colocalization of DNA decorated with CitH3 in *S. aureus*-induced ETs structures. WT and STING^-/-^ BMDNs were infected with *S. aureus* (MOI: 20) for 3 h and then fixed with 4% paraformaldehyde. DNA decorated with CitH3 within ETs was detected using a fluorescence microscope. DNA was stained with SYTOX green. The experiments were repeated 3 independent times.

Supplementary Figure 2 The effect of degradation of ETs with DNase I on producing cytokines after pulmonary *S. aureus* infection. WT mice were infected with *S. aureus* (1 × 10^8^ CFU/mouse, i.t.) and treated with DNase I (4000 U, i.t.) or diluent control at 2, 10, and 18 hpi (n = 4-6/group) and then were euthanized at 6 and 24 hpi. BALF and lung ho mogenates were collected for the analysis of indicated cytokines by ELISA. (A, D) IL-1β, (B, E) IL-6, (C, F) TNFα. Data are shown as the mean ± SEM. Data were pooled from 2 independent experiments. Significance calculated using two-way ANOVA with Bonferroni’s multiple comparison test.

## Abbreviations

*S. aureus*, *Staphylococcus aureus*, STING: stimulator of interferon genes, MRSA: methicillin-resistant *Staphylococcus aureus*, ET: extracellular trap, cfDNA: cell-free DNA, CitH3: citrullinated histone H3, MPO: myeloperoxidase, NE: neutrophil elastase, ROS: reactive oxygen species, ERK1/2: extracellular signal-regulated kinase 1 and 2, LPS: lipopolysaccharide, PRRs: pattern recognition receptors, TLR: Toll-like receptor, GSDMD: Gasdermin D, PAD4: peptidylarginine deiminase 4, CytD: cytochalasin D, *i. p.*: intraperitoneally, *i. t.*: intranasally, PBS: phosphate buffered saline, TSB: tryptic soy broth, MOI: multiplicity of infection, BMDM: bone marrow-derived macrophage, BMDN: bone marrow-derived neutrophil.
