# Supplementary figures and images for: Stimulator of Interferon Genes (STING) Promotes Staphylococcus aureus-Induced Extracellular Traps Formation via the ROS-ERK Signaling Pathway
Source: Front Cell Dev Biol. 2022 Mar 23;10:836880. doi: 10.3389/fcell.2022.836880 (PMC8984202; doi:10.3389/fcell.2022.836880)

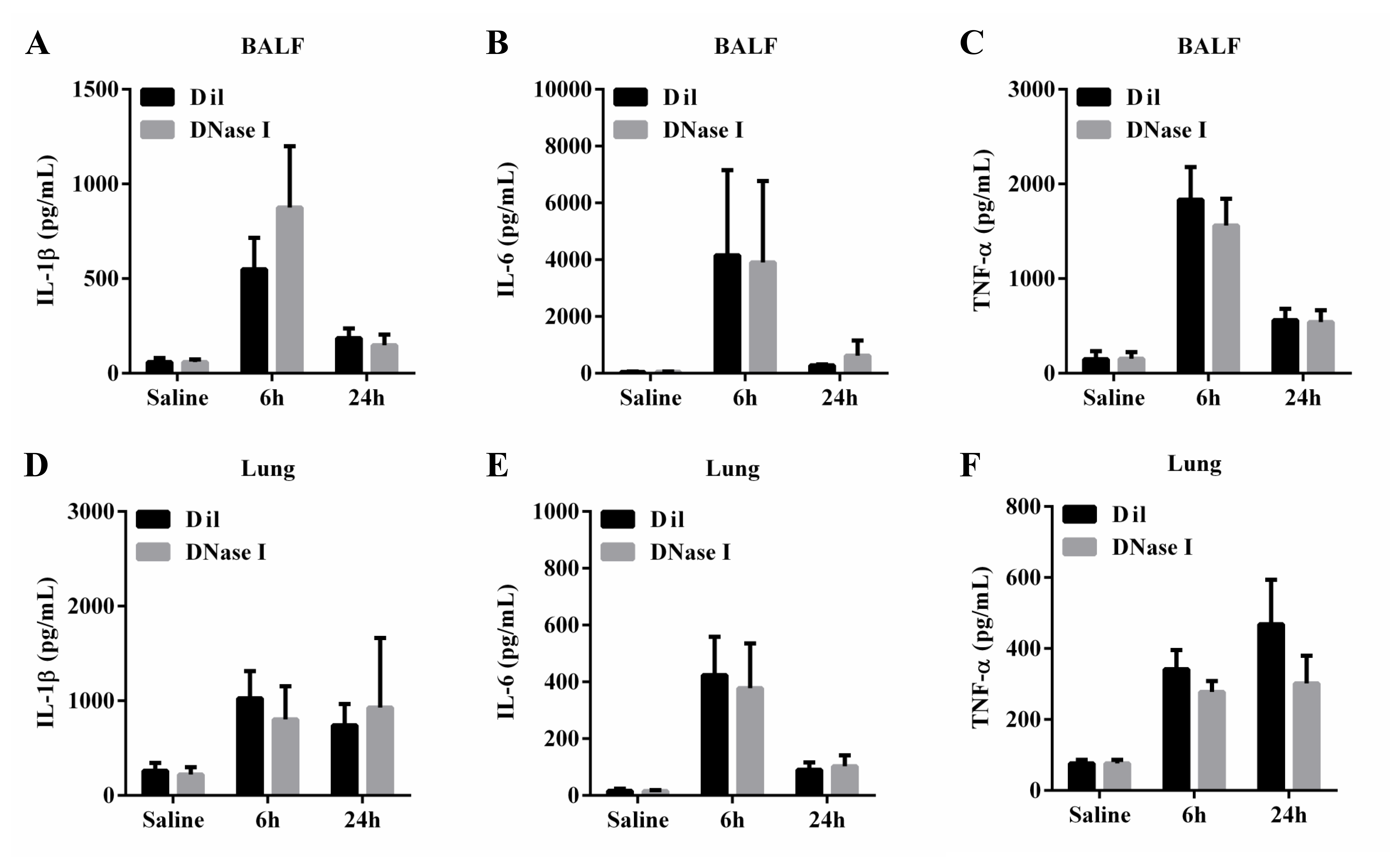

Supplement: Supplementary file 1 [file Image2.tif]

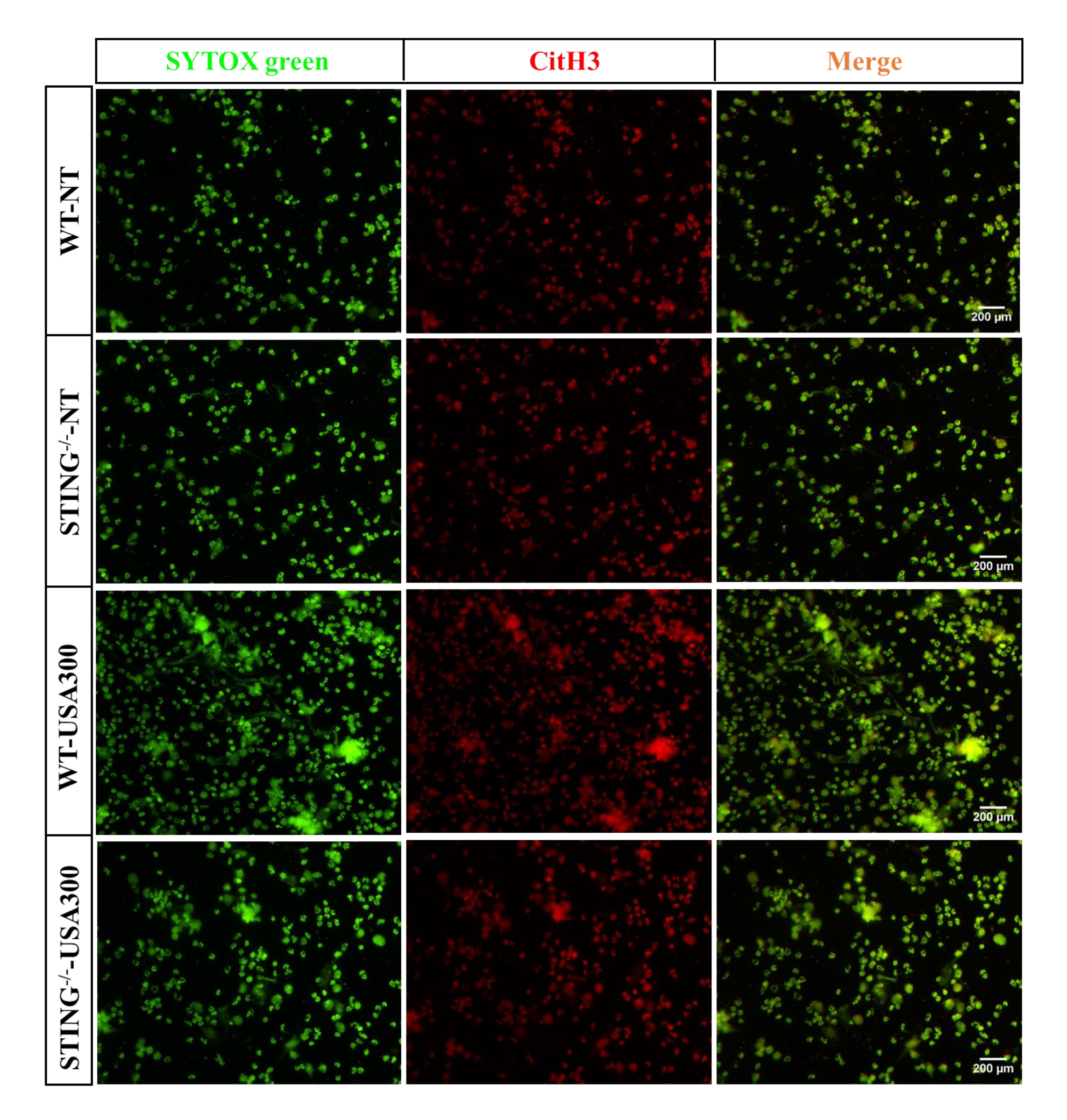

Supplement: Supplementary file 2 [file Image1.tif]
