# Supplementary material for: Stimulator of Interferon Genes (STING) Promotes Staphylococcus aureus-Induced Extracellular Traps Formation via the ROS-ERK Signaling Pathway
Source: Front Cell Dev Biol. 2022 Mar 23;10:836880. doi: 10.3389/fcell.2022.836880 (PMC8984202; doi:10.3389/fcell.2022.836880)

**
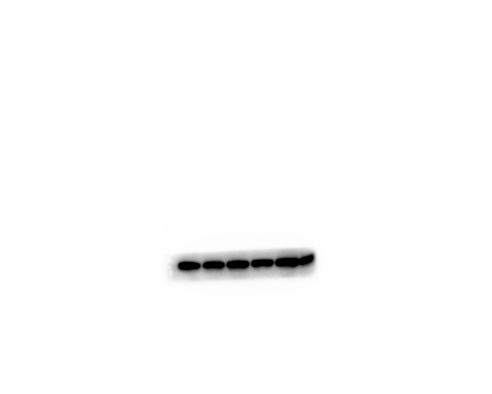

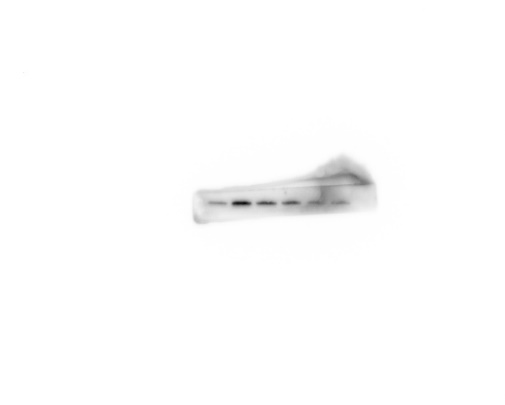
Figure 1C**

CitH3 H3

GAPDH


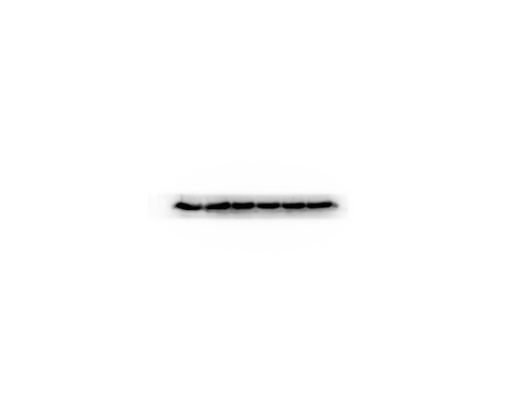


**Figure 1D** (lanes 1-4)


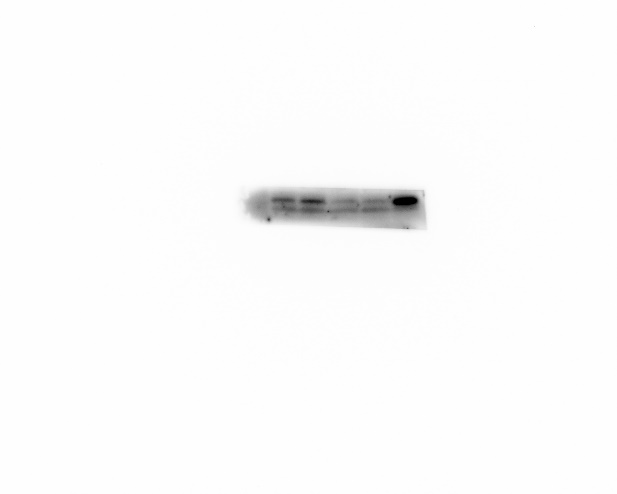
CitH3 (nucleus) H3 (nucleus)


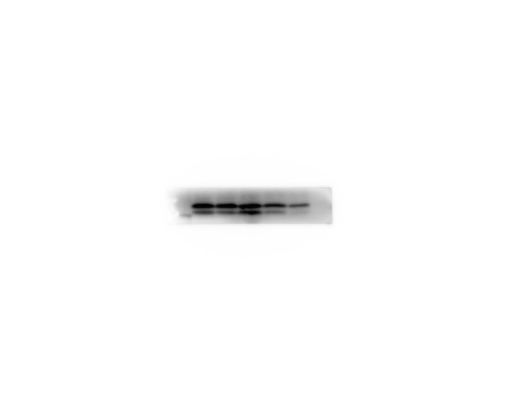


CitH3 (cytoplasm) GAPDH


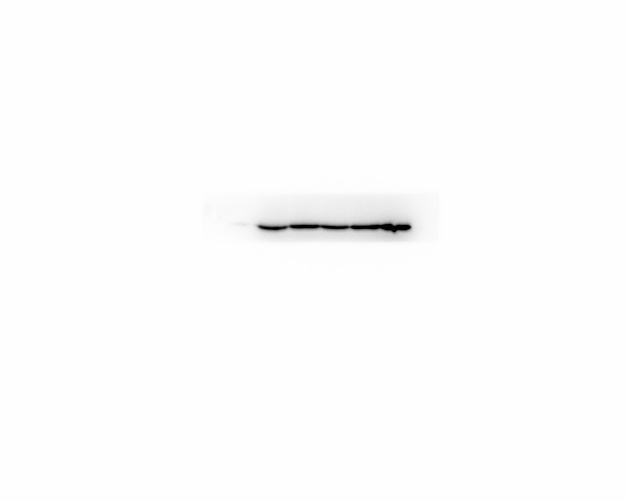

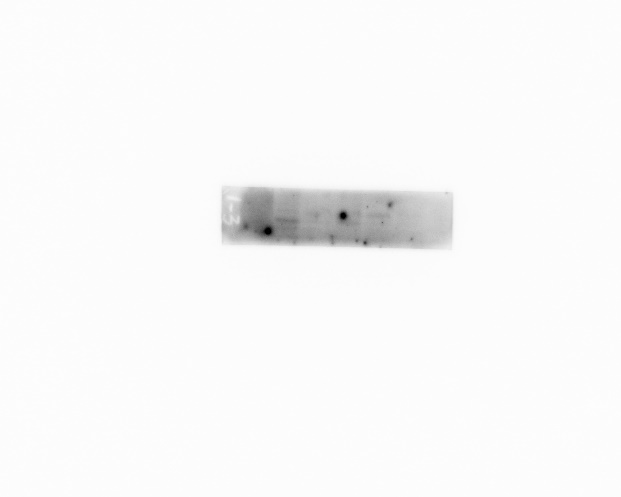


**Figure 4B**


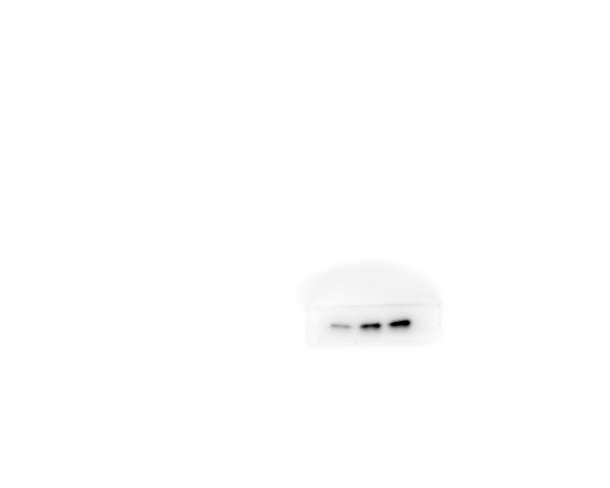
p-ERK p-P38

**
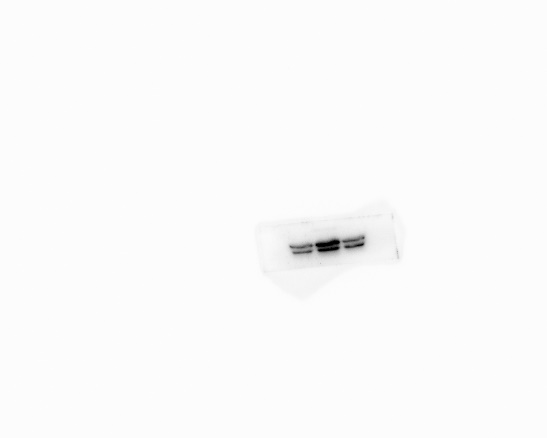
**

CitH3 H3

**
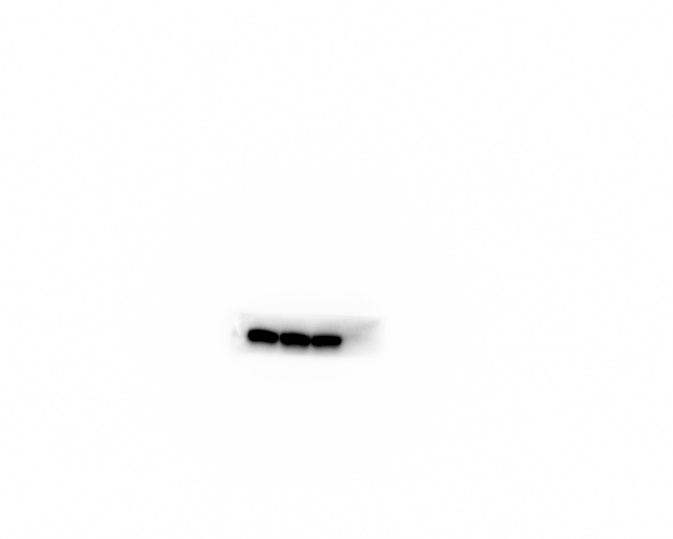

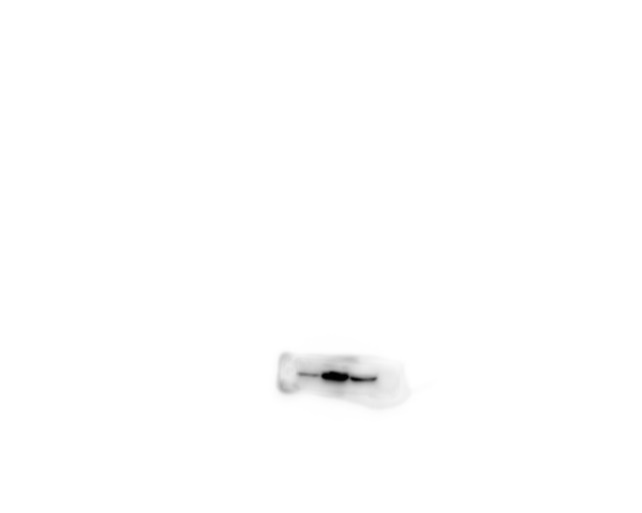
**


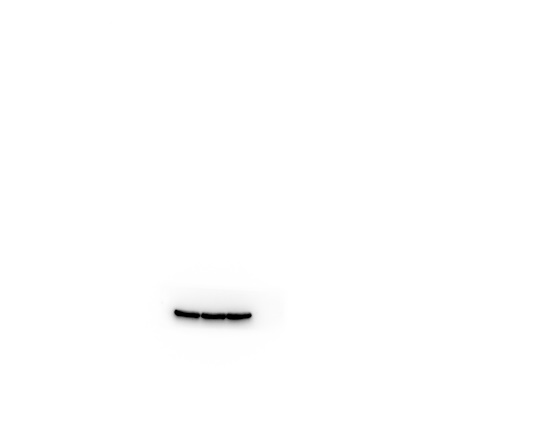
GAPDH

**Figure 4C**

p-ERK GAPDH


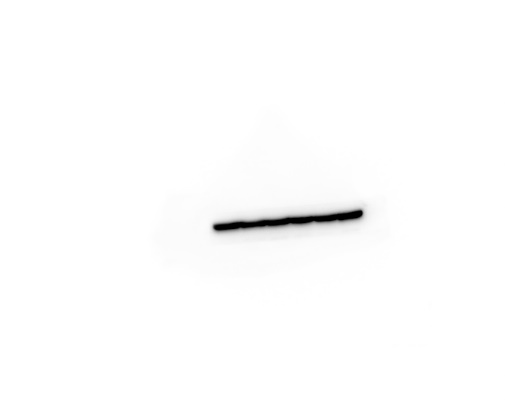
**
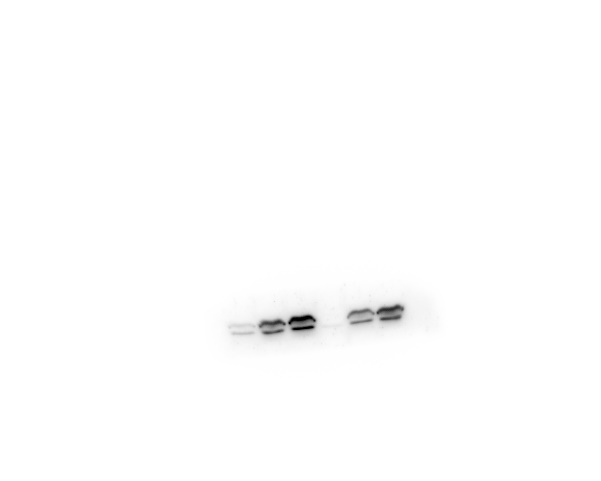
**

**Figure 4D** (lanes 1-8)


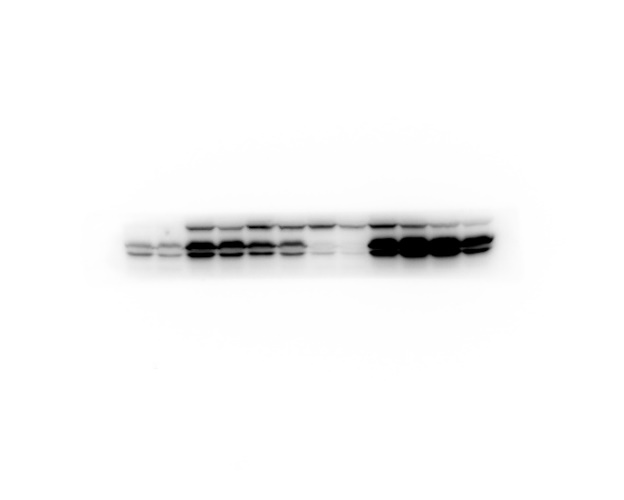

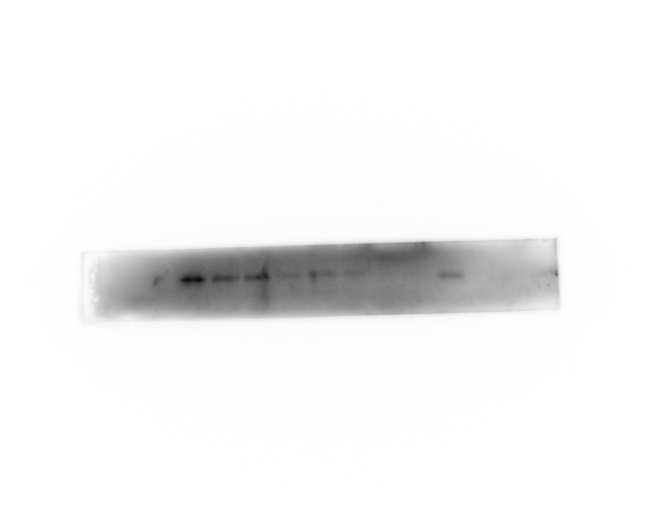
p-ERK CitH3


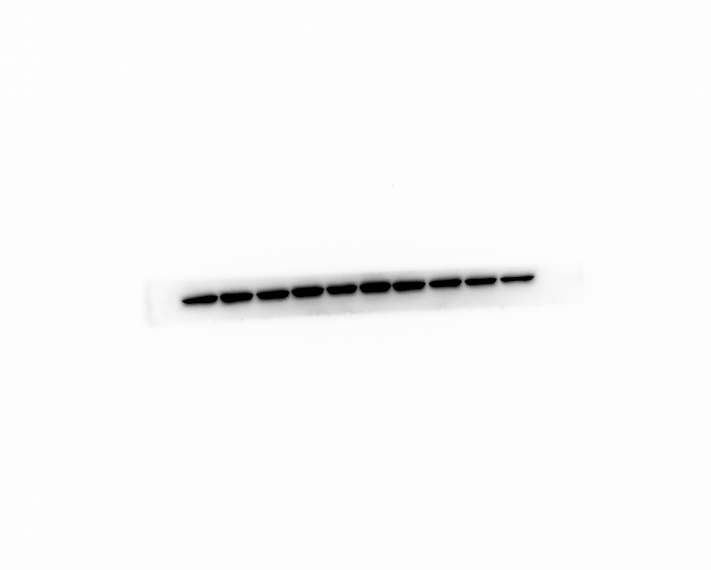

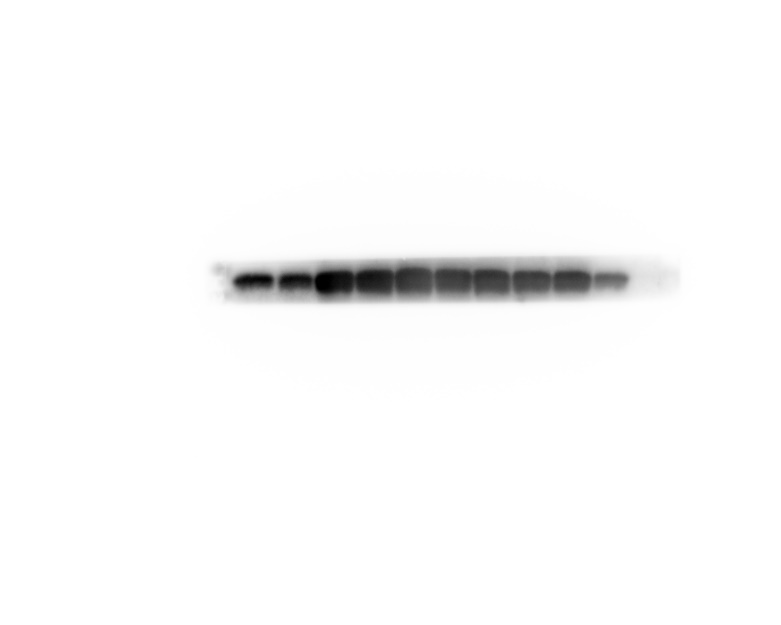
H3 GAPDH

**Figure 7C**


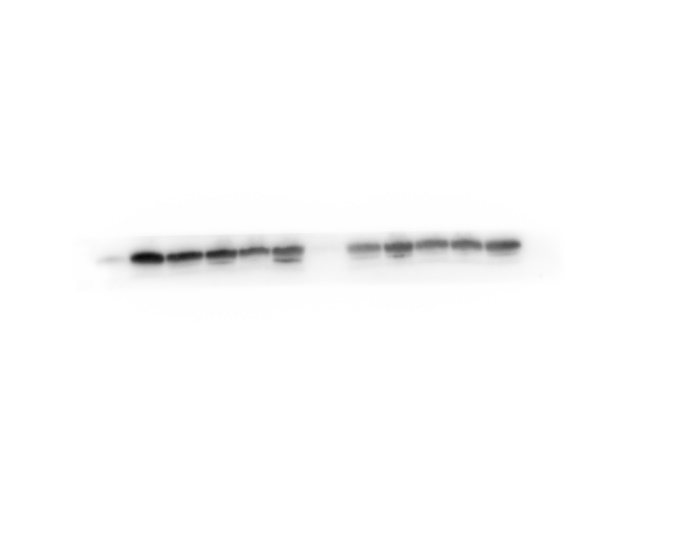

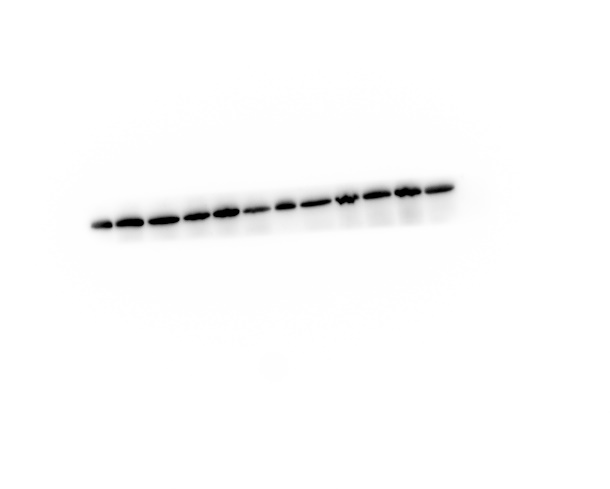
CitH3 GAPDH

**Figure 2A**


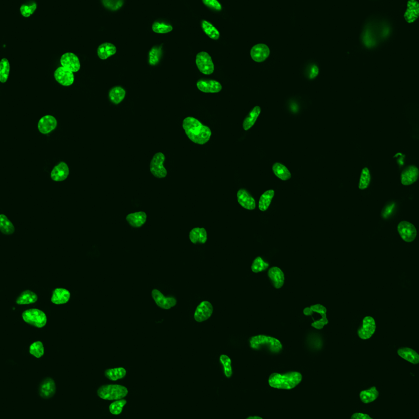

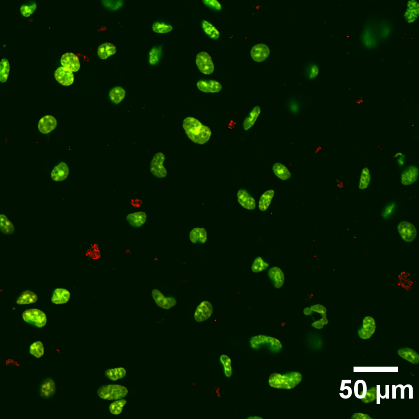

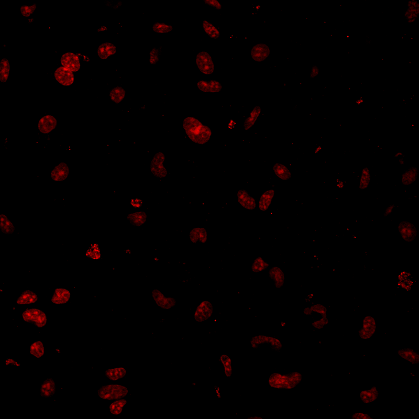

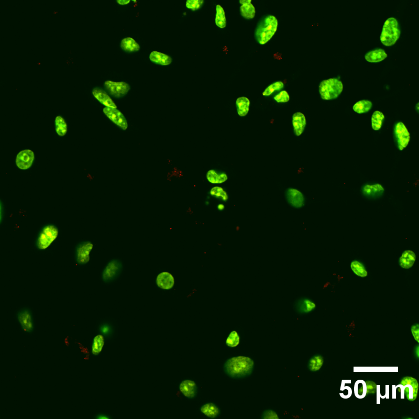

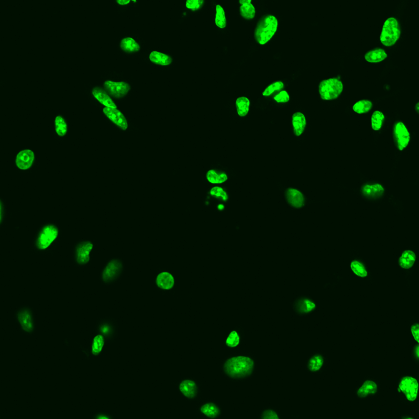

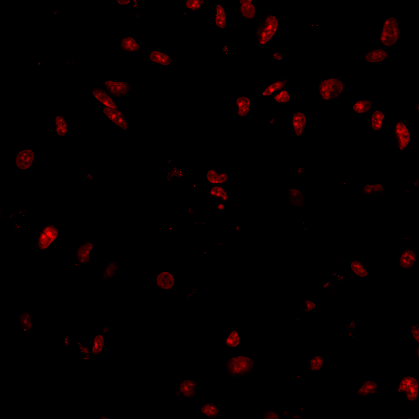


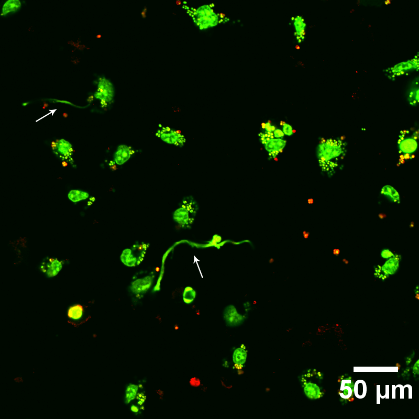

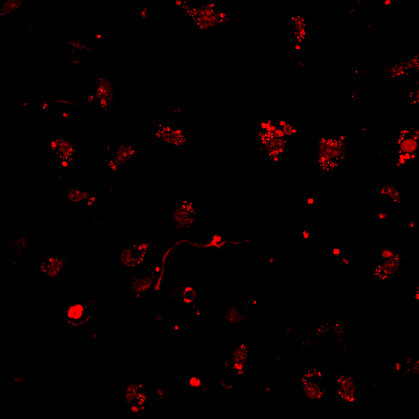

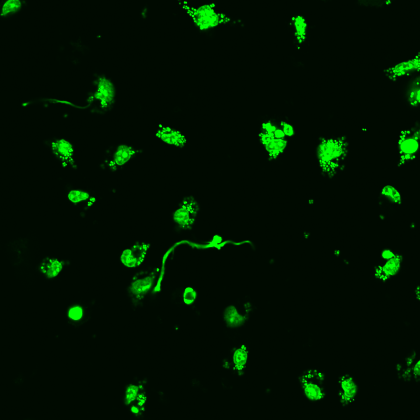


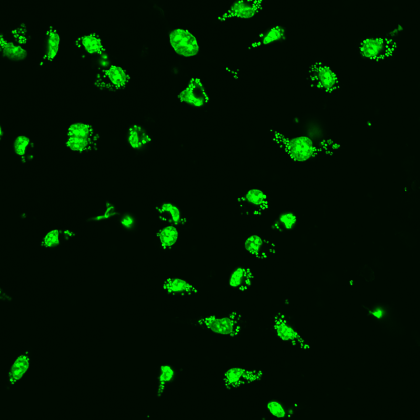

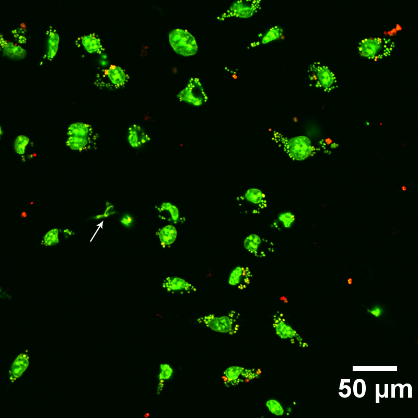

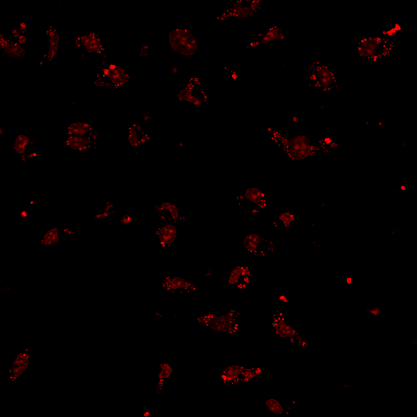


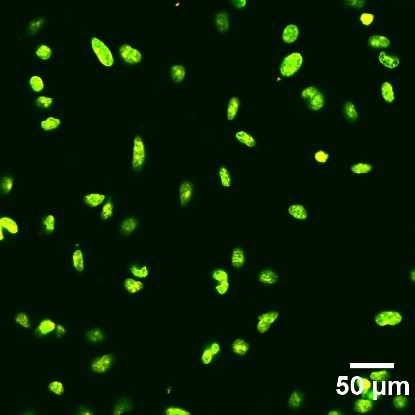

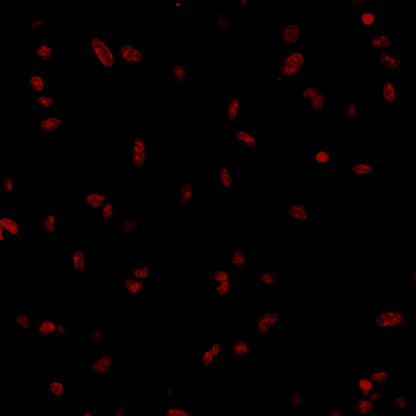

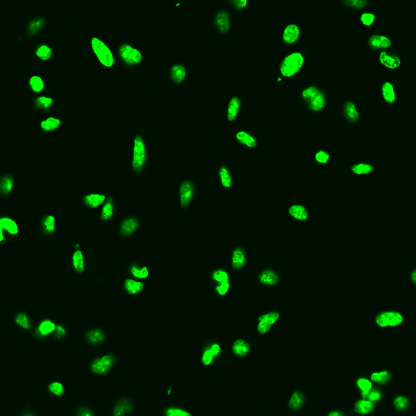
**Figure 2B**


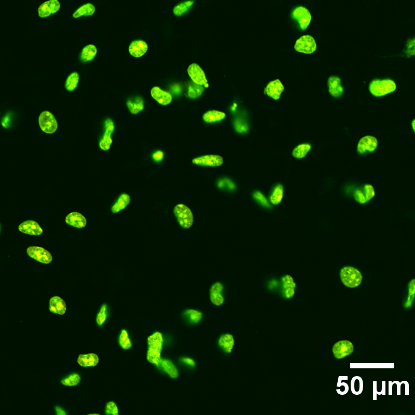

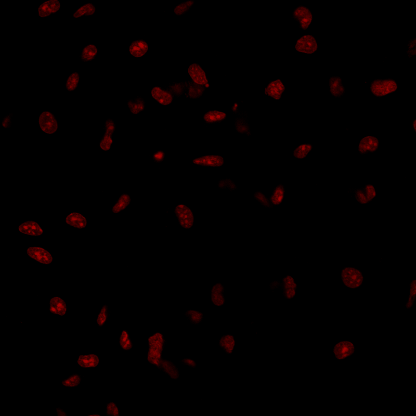

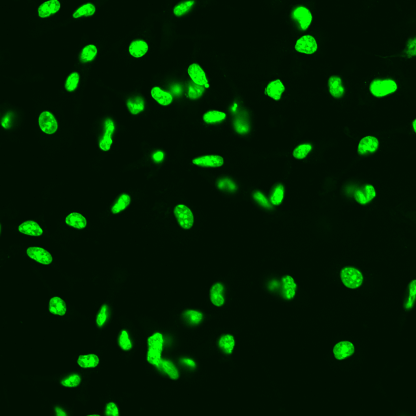


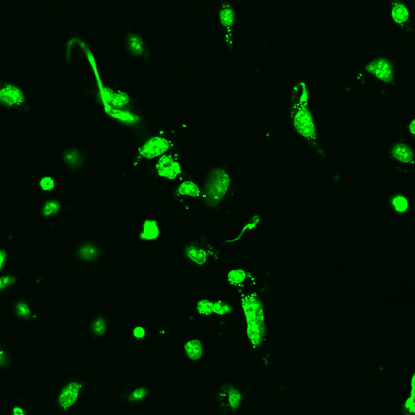


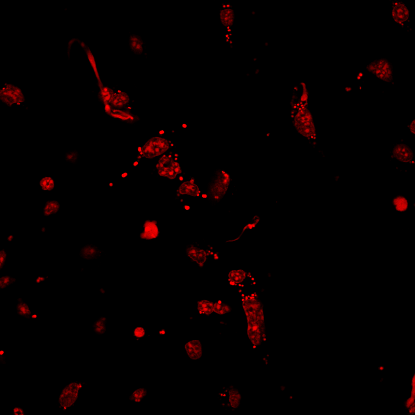


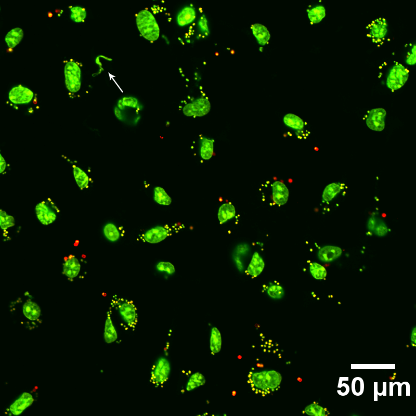

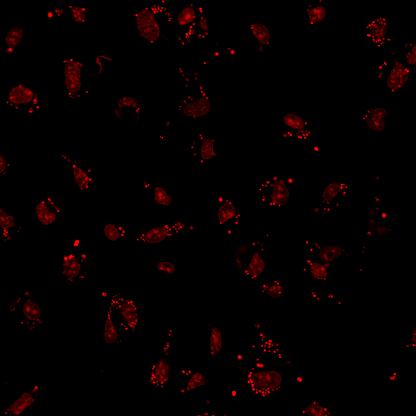

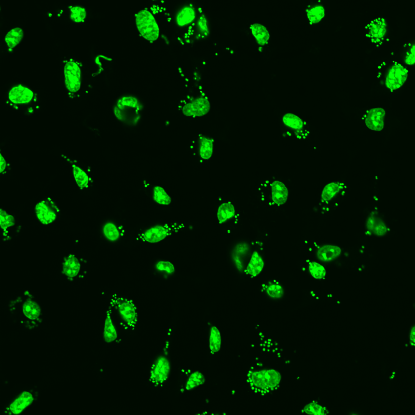


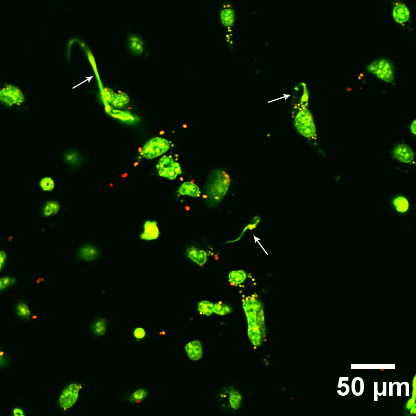


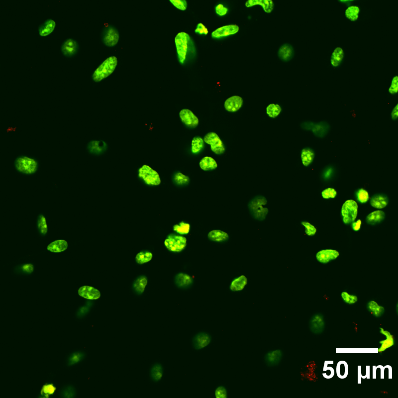

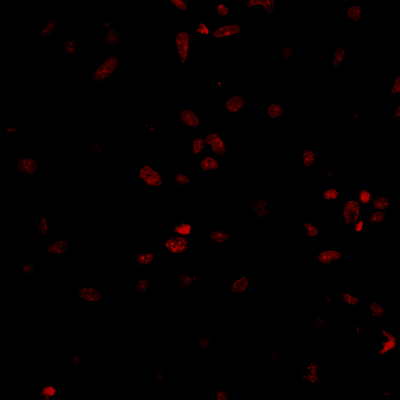

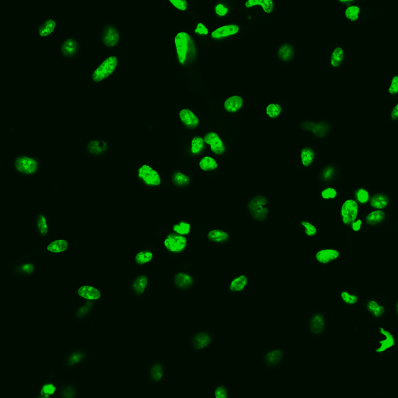
**Figure 3B**


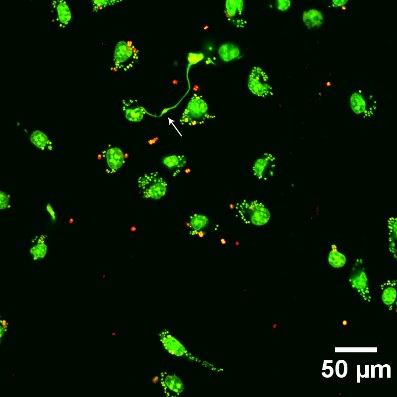

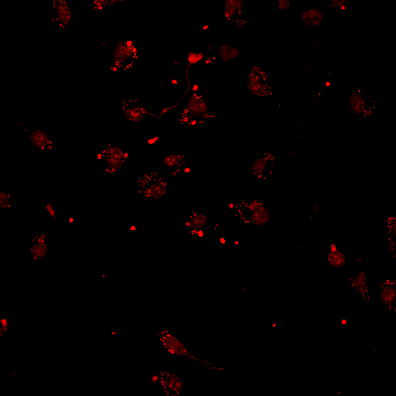

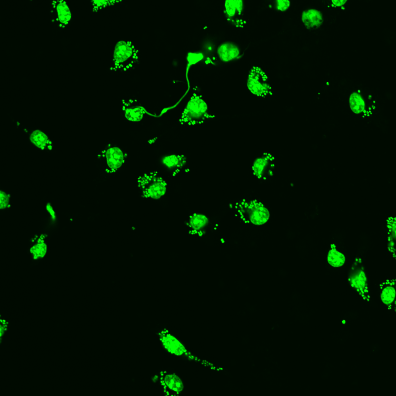


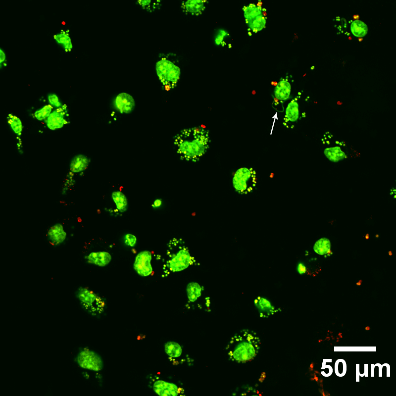

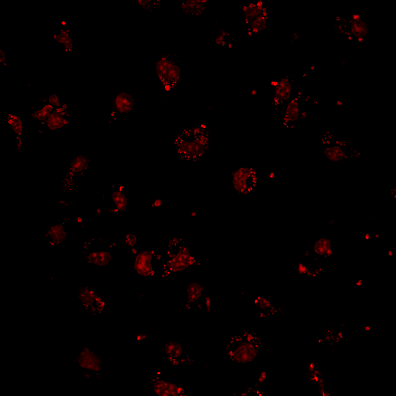

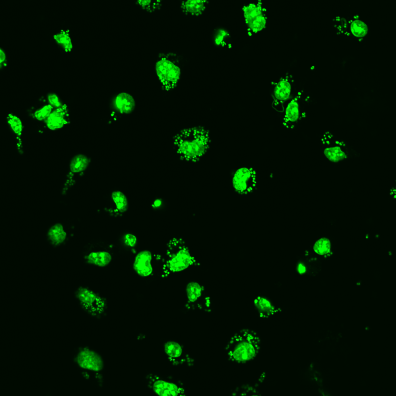


**Figure 3C**


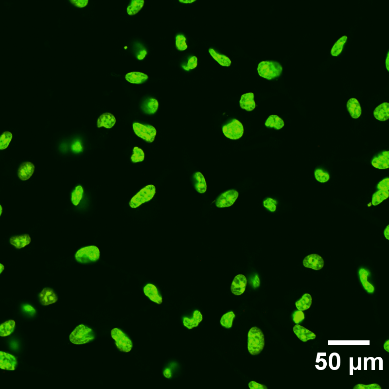

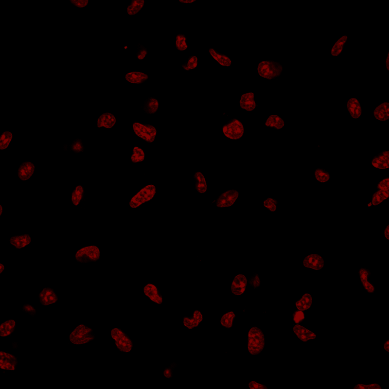

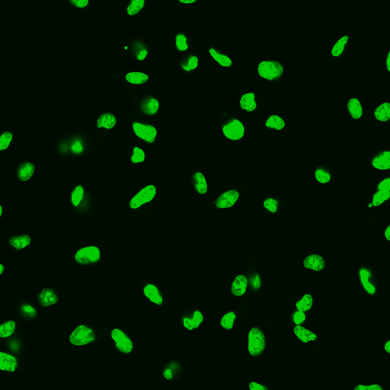


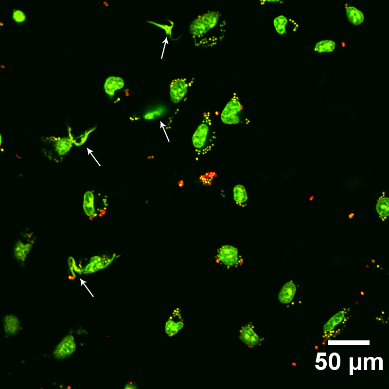

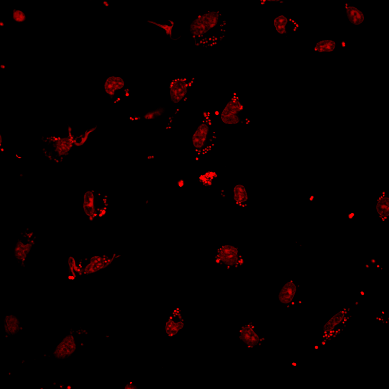

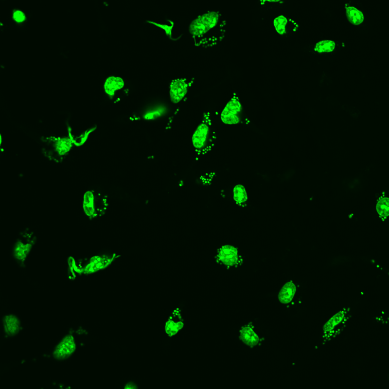


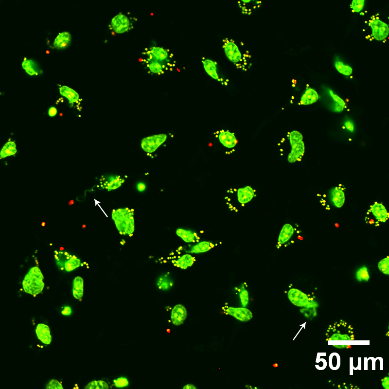

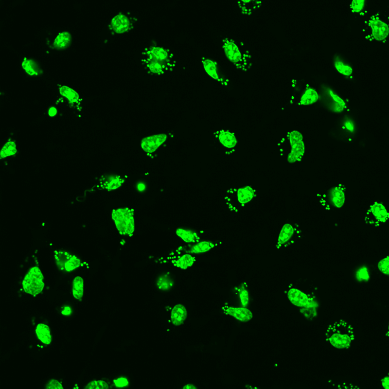

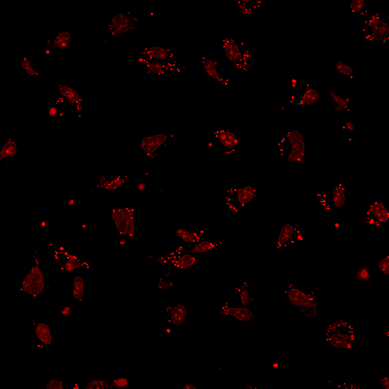


**Figure 7B**


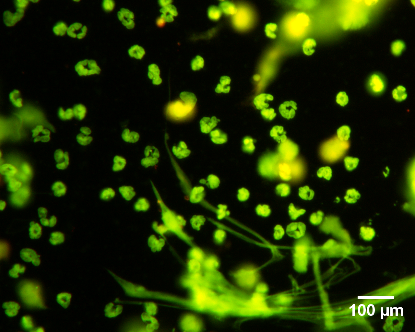
**
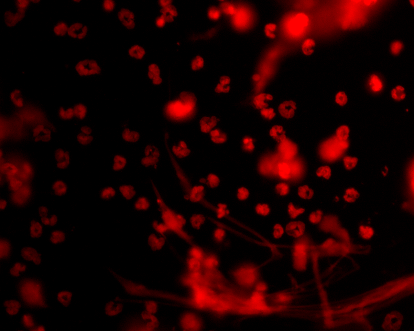
**
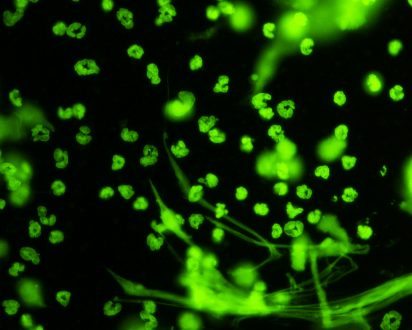


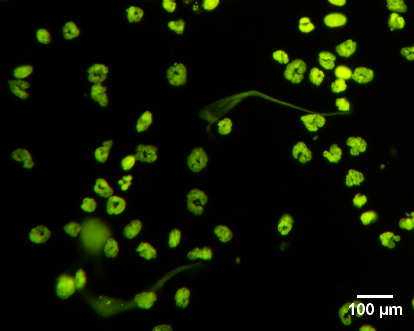

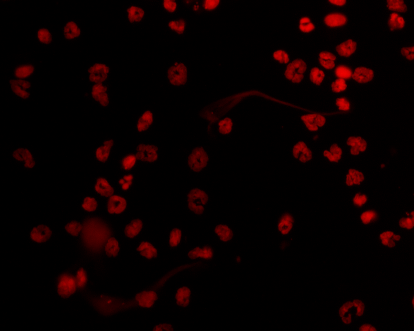

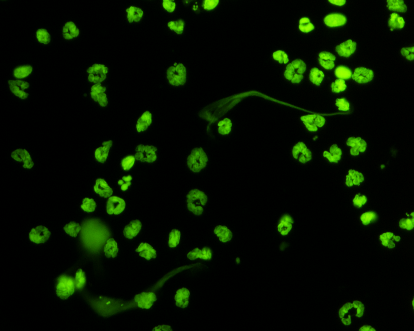

Supplement: Supplementary file 3 [file Table2.DOCX]
